# Systems Biology of Tissue-Specific Response to *Anaplasma phagocytophilum* Reveals Differentiated Apoptosis in the Tick Vector *Ixodes scapularis*

**DOI:** 10.1371/journal.pgen.1005120

**Published:** 2015-03-27

**Authors:** Nieves Ayllón, Margarita Villar, Ruth C. Galindo, Katherine M. Kocan, Radek Šíma, Juan A. López, Jesús Vázquez, Pilar Alberdi, Alejandro Cabezas-Cruz, Petr Kopáček, José de la Fuente

**Affiliations:** 1 SaBio. Instituto de Investigación en Recursos Cinegéticos IREC, CSIC-UCLM-JCCM, Ciudad Real, Spain; 2 Department of Veterinary Pathobiology, Center for Veterinary Health Sciences, Oklahoma State University, Stillwater, Oklahoma, United States of America; 3 Institute of Parasitology, Biology Centre, Academy of Sciences of the Czech Republic, České Budějovice, The Czech Republic; 4 Centro Nacional de Investigaciones Cardiovasculares (CNIC), Madrid, Spain; 5 Center for Infection and Immunity of Lille (CIIL), Université Lille Nord de France, Institut Pasteur de Lille, Lille, France; Genentech, UNITED STATES

## Abstract

*Anaplasma phagocytophilum* is an emerging pathogen that causes human granulocytic anaplasmosis. Infection with this zoonotic pathogen affects cell function in both vertebrate host and the tick vector, *Ixodes scapularis*. Global tissue-specific response and apoptosis signaling pathways were characterized in *I*. *scapularis* nymphs and adult female midguts and salivary glands infected with *A*. *phagocytophilum* using a systems biology approach combining transcriptomics and proteomics. Apoptosis was selected for pathway-focused analysis due to its role in bacterial infection of tick cells. The results showed tissue-specific differences in tick response to infection and revealed differentiated regulation of apoptosis pathways. The impact of bacterial infection was more pronounced in tick nymphs and midguts than in salivary glands, probably reflecting bacterial developmental cycle. All apoptosis pathways described in other organisms were identified in *I*. *scapularis*, except for the absence of the Perforin ortholog. Functional characterization using RNA interference showed that Porin knockdown significantly increases tick colonization by *A*. *phagocytophilum*. Infection with *A*. *phagocytophilum* produced complex tissue-specific alterations in transcript and protein levels. In tick nymphs, the results suggested a possible effect of bacterial infection on the inhibition of tick immune response. In tick midguts, the results suggested that *A*. *phagocytophilum* infection inhibited cell apoptosis to facilitate and establish infection through up-regulation of the JAK/STAT pathway. Bacterial infection inhibited the intrinsic apoptosis pathway in tick salivary glands by down-regulating Porin expression that resulted in the inhibition of Cytochrome c release as the anti-apoptotic mechanism to facilitate bacterial infection. However, tick salivary glands may promote apoptosis to limit bacterial infection through induction of the extrinsic apoptosis pathway. These dynamic changes in response to *A*. *phagocytophilum* in *I*. *scapularis* tissue-specific transcriptome and proteome demonstrated the complexity of the tick response to infection and will contribute to characterize gene regulation in ticks.

## Author Summary

The continuous human exploitation of environmental resources and the increase in human outdoor activities, which have allowed for the contact with arthropod vectors normally present in the field, has promoted the emergence and resurgence of vector-borne pathogens. Among these, *Anaplasma phagocytophilum* is an emerging bacterial pathogen transmitted to humans and other vertebrate hosts by ticks as they take a blood meal that causes human granulocytic anaplasmosis in the United States, Europe and Asia, with increasing numbers of affected people every year. Tick response to pathogen infection has been only partially characterized. In this study, global tissue-specific response and apoptosis signaling pathways were characterized in tick nymphs and adult female midguts and salivary glands infected with *A*. *phagocytophilum* using a systems biology approach combining transcriptomics and proteomics. The results demonstrated dramatic and complex tissue-specific response to *A*. *phagocytophilum* in the tick vector *Ixodes scapularis*, which reflected pathogen developmental cycle and the impact on tick apoptosis pathways. These dynamic changes in response to *A*. *phagocytophilum* in *I*. *scapularis* tissue-specific transcriptome and proteome demonstrated the complexity of the tick response to infection and contributes information on tick-pathogen interactions and for development of novel control strategies for pathogen infection and transmission.

## Introduction

*Anaplasma phagocytophilum* (Rickettsiales: Anaplasmataceae) is an emerging zoonotic pathogen transmitted by *Ixodes* ticks of which the major vector species are *I*. *scapularis* in the US and *I*. *ricinus* in Europe [1]. This intracellular bacterium infects tick midguts [2] and salivary glands [3] and vertebrate host granulocytes causing human, canine and equine granulocytic anaplasmosis and tick-borne fever of ruminants [4–8]. Human granulocytic anaplasmosis is the second most common tick-borne disease in the United States and tick-borne fever is an established and economically important disease of sheep in Europe [8, 9].

The molecular mechanisms used by *A*. *phagocytophilum* to infect and multiply within vertebrate hosts including the inhibition of neutrophil apoptosis have been well characterized [5, 10–14]. *Anaplasma* infection in the tick vector has been shown to modulate gene expression and tick proteins have been identified that interfere with bacterial acquisition and/or transmission [15]. However, little information is available on the impact of pathogen infection at both transcriptome and proteome levels and the molecular pathways affected by *A*. *phagocytophilum* to establish infection in ticks. Recently, Ayllón et al. [16] demonstrated that *A*. *phagocytophilum* infection inhibits tick intrinsic apoptosis pathway resulting in increased infection and Severo et al. [6] defined a role for ubiquitination during bacterial colonization of tick cells. However, as shown for other tick-pathogen models [17], information is not available on the tick tissue-specific responses to *A*. *phagocytophilum* infection. These facts underline the importance of defining strategies by which these bacteria establish infection in the tick vector.

As recently shown for *Drosophila melanogaster*, arthropod transcriptomes and proteomes are dynamic, with each developmental stage and organ presenting an ensemble of transcripts and proteins that give rise to substantial diversity in their profile [18]. Characterization of tissue-specific responses and cellular pathways in ticks in response to infection with *A*. *phagocytophilum* by use of high-throughput omics technologies is essential for understanding tick-pathogen interactions and to provide targets for development of novel control strategies for both vector infestations and pathogen infection/transmission [15, 19, 20]. However, the application of a systems biology approach to the study of non-model organisms such as tick-pathogen interactions poses challenges including the analysis of large datasets in order to extract biologically relevant information and interpret changes in gene expression in relation to simultaneous changes in the proteome [21–23]. The *I*. *scapularis* genome is the only tick genome sequenced (GenBank accession ABJB010000000) but limitations in genome assembly and annotation add additional complexity to the characterization of the molecular events at the tick-pathogen interface [23–25]. Thus, the design of experiments combining tick transcriptomics and proteomics require the integration of these different datasets to identify relevant biological processes and molecules. This challenge can be addressed by assessing global transcriptome and proteome changes and studying specific pathways such as immune response and apoptosis that are important for pathogen infection and transmission by ticks.

In this study, we characterized global tissue-specific response and apoptosis signaling pathways in *I*. *scapularis* infected with *A*. *phagocytophilum*. Apoptosis was selected for pathway-focused analysis due to its role in *A*. *phagocytophilum* infection of tick cells [16]. Nymphs and female midguts and salivary glands were selected for this analysis using a systems biology approach combining transcriptomics and proteomics data. These tick developmental stages and tissues were selected for this study because nymphs are the main vectors for pathogen transmission to humans and animals while midguts and salivary glands play a major role during pathogen acquisition, multiplication and transmission [15, 26]. The hypotheses addressed in this study included that tick tissue-specific response to infection reflects pathogen developmental cycle and *A*. *phagocytophilum* infection impacts on tick apoptosis pathways in a tissue-specific manner. The results showed that *A*. *phagocytophilum* infection results in complex and dramatic tissue-specific changes of the tick transcriptome and proteome and further extended our understanding of the role of selected biological pathways during bacterial infection and multiplication in the tick vector.

## Results and Discussion

### *A*. *phagocytophilum* infection results in complex and dramatic tissue-specific changes of the tick transcriptome and proteome

**Fig 1:**
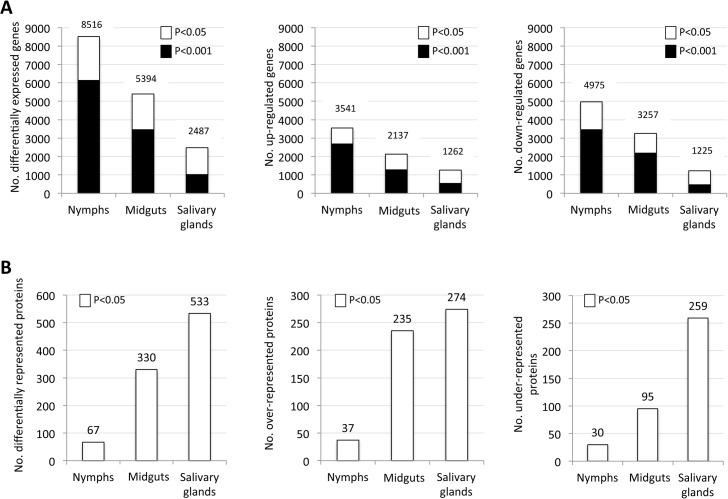
Tissue-specific differentially expressed genes and represented proteins in response to *A*. *phagocytophilum* infection. Tissue-specific differences in tick response to pathogen infection were found at both mRNA and protein levels. (A) Number of differentially expressed genes (total, up-regulated and down-regulated) in infected nymphs, adult female midguts and salivary glands. (B) Number of differentially represented proteins (total, over-represented and under-represented) in infected nymphs, adult female midguts and salivary glands (-2>Zq>2).

*A*. *phagocytophilum*, as other obligate intracellular bacteria, evolved to manipulate host cells to establish infection [27]. Pathogen survival requires the alteration of cell native functions to allow infection, multiplication and transmission. The impact of pathogen infection on host cell function is reflected by changes in the transcriptome and proteome, something that was characterized here at tissue-specific level in ticks infected with *A*. *phagocytophilum*. Two independent samples were collected and processed for each tick developmental stage and tissue. After RNAseq, 2.1–4.1 Gbp (Ave±SD; 2.8±0.6) high quality reads were obtained for nymph, adult female midgut and salivary gland samples in infected and uninfected ticks with 101±2 bp average read length and less than 10% (0–8%) variation between replicates (S1 Table). These reads were aligned to the *I*. *scapularis* reference genome using TopHat and resulted in 16083, 12651 and 11105 gene transcripts in nymph, midgut and salivary gland samples, respectively with 16293 (99–231014) bp average length and 48±6%GC content (S2 Table). The number of unique gene transcripts mapped among all samples (17503) represented 85% of the predicted 20486 protein-coding genes in the *I*. *scapularis* genome [28]. Of the mapped transcripts, 8516 (53%), 5394 (43%) and 2487 (22%) were differentially expressed in response to *A*. *phagocytophilum* infection in nymph, midgut and salivary gland samples, respectively (P<0.05; Fig 1A and S2 Table). Probably due to the fact that whole internal organs were analyzed in nymphs, the number of differentially expressed genes in the nymphs was similar to the total number of differentially expressed genes in adult female midguts and salivary glands together (Fig 1A), suggesting that other tissues did not contribute much to the transcriptome in nymphs. However, differences were observed in the number of up- and down-regulated genes in different samples with a higher number of down-regulated genes in nymphs and midguts while in salivary glands the number of up- and down-regulated genes was similar (Fig 1A). We used P<0.05 for differential gene expression analysis, but a high proportion of the differentially expressed genes were significantly different between infected and uninfected samples at P<0.001 (Fig 1A), providing additional support for the transcriptomics data.

Proteomics analysis resulted in the identification of 7418 unique proteins, representing 36% of the predicted proteins encoded by the *I*. *scapularis* genome [28]. The number of proteins identified in nymphs (738) was lower than the number of proteins identified in midguts (4195) and salivary glands (6324), but the fraction of proteins matching *I*. *scapularis* identifications was similar between samples (53–66%), supporting that sampling did not affect protein assignations. Of the identified proteins, 67, 330 and 533 were differentially represented in response to *A*. *phagocytophilum* infection in nymphs, adult female midguts and salivary glands, respectively (Fig 1B and S3 Table).

Despite the difference between the number of mapped transcripts and proteins due to the lower resolution of protein identification when compared to transcriptomics [29], the coverage of the tick proteome reported here was high for ticks [23]. Similar to the transcriptomics analysis, differences were observed in the number of over- and under-represented proteins in different samples with a similar number of over-represented proteins in midguts and salivary glands but 2.7-fold more under-represented proteins in the salivary glands than in the midguts (Fig 1B).

At the individual mRNA and protein levels, a moderate (R^2^ = 0.4) correlation was obtained for the entire dataset but for genes and proteins highly up- and down-regulated/represented correlation did not exist (S1 Fig). The discrepancy between mRNA and protein levels could be explained by delay between mRNA and protein accumulation which requires sampling at different time points and/or the role for post-transcriptional and post-translational modifications in tick tissue-specific response to *A*. *phagocytophilum* infection. For example, apoptosis is often regulated at the post-transcriptional level [30].

The analysis of the total number of differentially expressed genes and represented proteins identified in tick samples highlighted dramatic tissue-specific differences in tick response to *A*. *phagocytophilum* infection. To characterize the complexity of the effect of pathogen infection on tick tissues, gene and protein ontology (GO) analyses were conducted to allow for a better characterization of tissue-specific differences in response to infection.

The GO analysis revealed that as expected for the incomplete annotation of the *I*. *scapularis* genome, many of the genes and proteins were assigned to unknown (“Others”) biological process (BP) or molecular function (MF) (S2 and S3 Figs). Nevertheless, cellular process, metabolic process and regulation were the most represented BP while catalytic activity and binding were the most represented MF in all tissues for both transcripts and proteins (S2 and S3 Figs). However, tissue-specific differences were also found that were more evident at the mRNA than at the protein level (S2 and S3 Figs), thus illustrating the complexity of the tick tissue-specific response to *A*. *phagocytophilum* infection.

### Tissue-specific response to infection reflects bacterial life cycle in ticks

**Fig 2:**
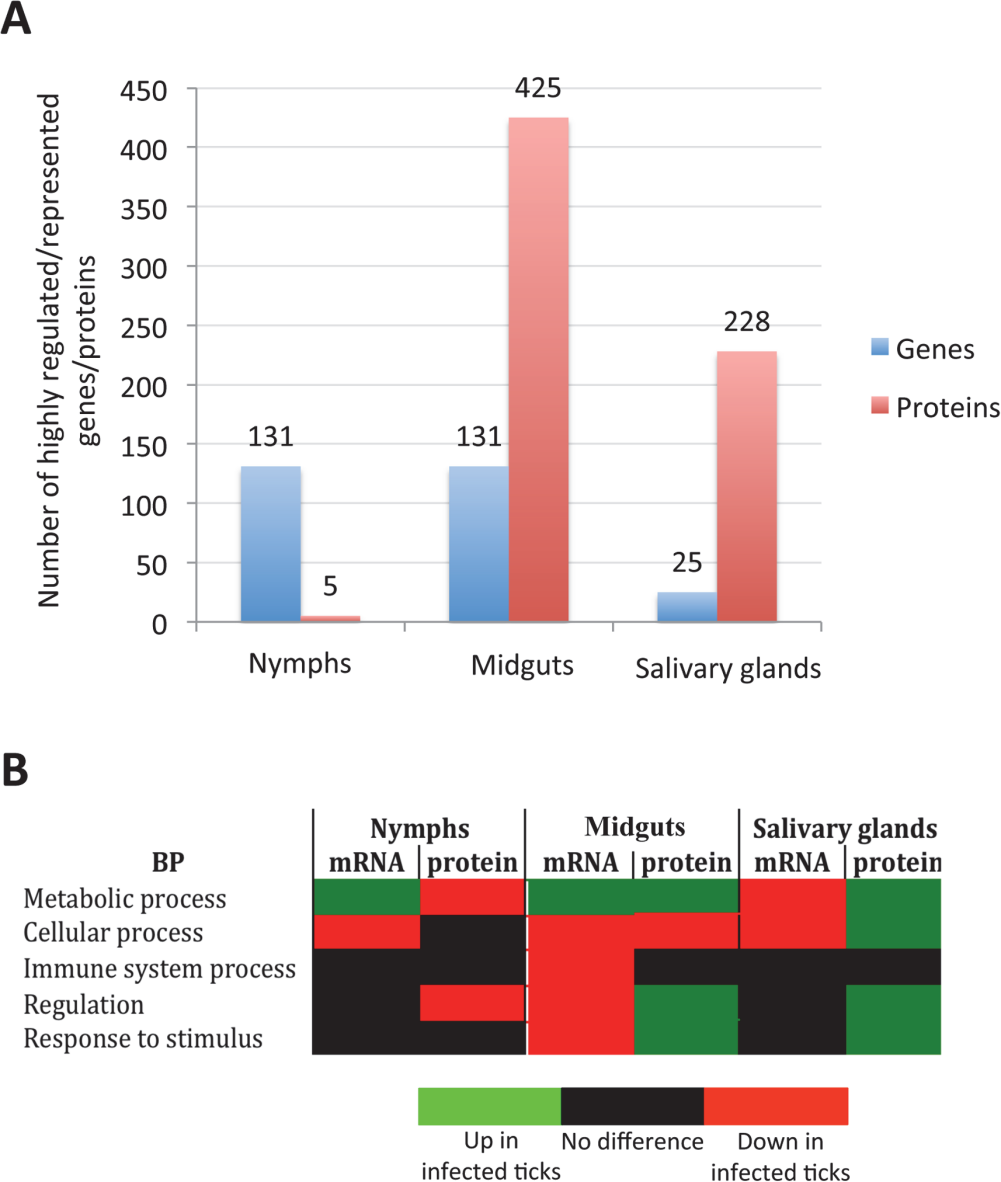
Tissue-specific effect of *A*. *phagocytophilum* infection on tick biological processes. The impact of bacterial infection on tick gene expression was more pronounced in nymphs and adult female midguts than in adult female salivary glands, probably reflecting pathogen developmental cycle. (A) Top differentially expressed genes were selected as those with more than 50-fold (log2 normalized fold change > 5.64) difference between infected and uninfected ticks. Top differentially represented proteins were selected as those with more than 5-fold (log2 normalized fold change > 2.32) difference between infected and uninfected ticks. (B) Predominant effect of bacterial infection on tick BPs. The number of top differentially expressed/represented genes/proteins in selected BPs was normalized against the total number of genes/proteins highly expressed/represented in nymphs, adult female midguts and salivary glands to select the predominant effect of bacterial infection on tick BPs.

The GO analysis is redundant because the same gene/protein may participate in more than one BP or MF, a problem that can be overcome in part by considering as one category in the analysis highly expressed/represented genes/proteins to reduce the number of entries per category. The analysis of tick genes/proteins whose expression/representation varied in more than 50-fold/5-fold further illustrated the complexity of tissue-specific differences in response to infection (S4 and S5 Figs). The total number of highly differentially expressed/represented genes/proteins suggested that the impact of bacterial infection on tick gene expression was more pronounced in nymphs and adult female midguts than in adult female salivary glands (Fig 2A). The hypothesis is that tick tissue-specific response to infection reflects pathogen developmental cycle. In adult female midguts, bacterial infection had the highest impact on tick gene/protein expression/representation through down-regulation of immune system and/or cellular process and up-regulation of metabolic process, while in salivary glands the bacteria had a lower impact on cellular processes because it does not need to replicate and are ready for transmission to vertebrate hosts by feeding ticks (Fig 2B). However, as a dynamic process, bacterial replication at earlier developmental stages may also affect cellular processes in salivary glands. These results reflected *A*. *phagocytophilum* developmental cycle in adult female tick tissues in which the intracellular reticulated, replicative form more abundant in midgut cells converts to the non-dividing infectious dense-core form more abundant in the salivary glands where bacterial transcription and translation are more active than replication [26].

### All apoptosis pathways described in other organisms were putatively identified in *I*. *scapularis* and are affected in response to *A*. *phagocytophilum* infection

Apoptosis is one of the pathways affected by intracellular bacteria such as *A*. *phagocytophilum* to establish infection in vertebrate host cells [31] and preliminary results suggested a role for apoptosis during infection of tick cells [16]. Our hypothesis is that *A*. *phagocytophilum* infection impacts on tick apoptosis pathways in a tissue-specific manner. To test this hypothesis, putative apoptosis pathway genes were annotated and then used to characterize tissue-specific differential gene/protein expression/representation in response to bacterial infection in combination with functional analyses.

**Fig 3:**
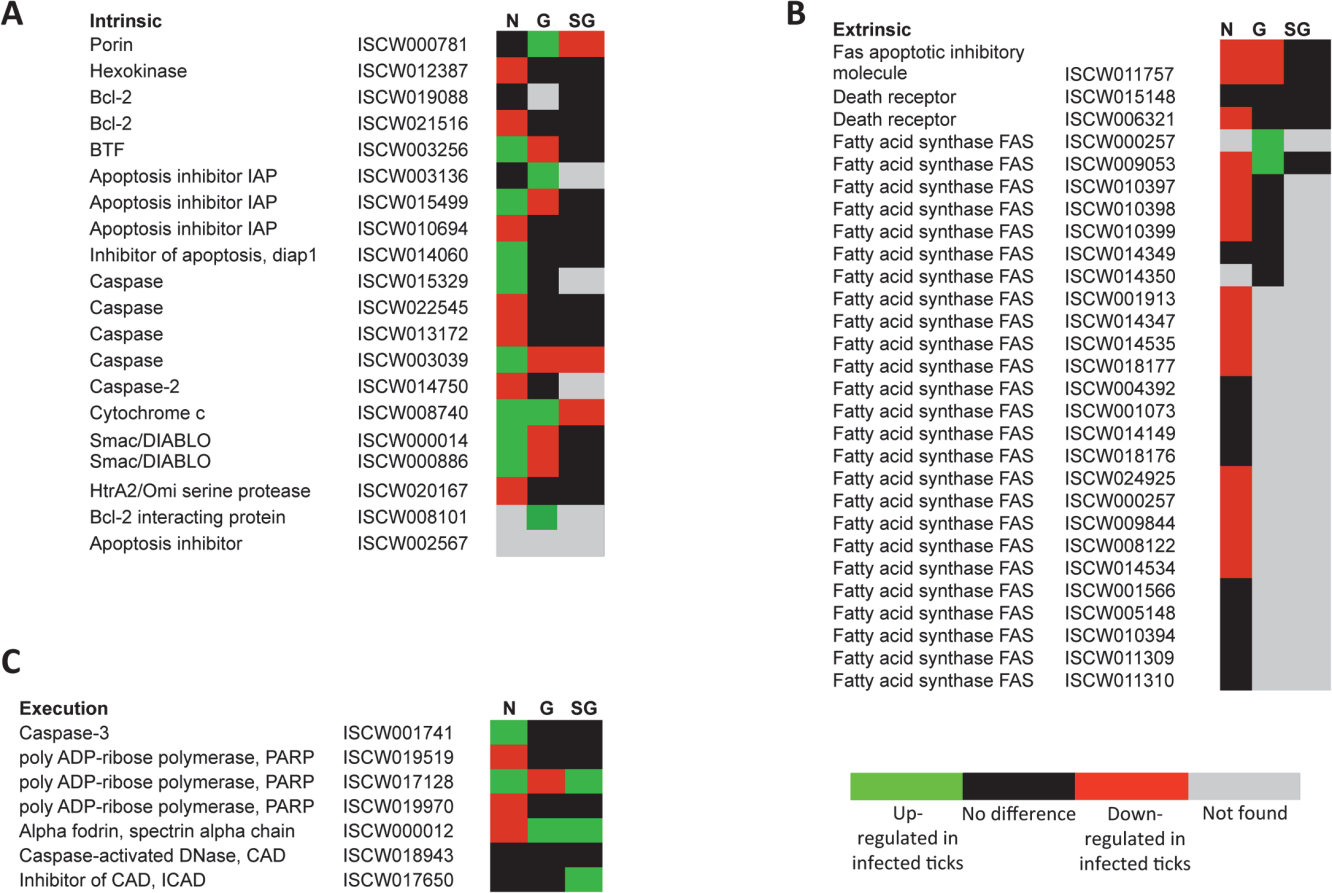
Annotation of intrinsic, extrinsic and execution apoptosis pathway genes and expression in response to *A*. *phagocytophilum* infection. Tissue-specific differences were found in the apoptosis pathway genes response to *A*. *phagocytophilum* infection. (A) Intrinsic pathway. (B) Extrinsic pathway. (C) Execution pathway. For annotation, gene identifiers were obtained from VectorBase (www.vectorbase.org) and compared to the corresponding pathways in *Drosophila melanogaster*, *Anopheles gambiae*, *Aedes aegypti* and *Homo sapiens*. Differential expression (P<0.05) is shown for tick nymphs (N) and adult female midguts (G) and salivary glands (SG).

**Fig 4:**
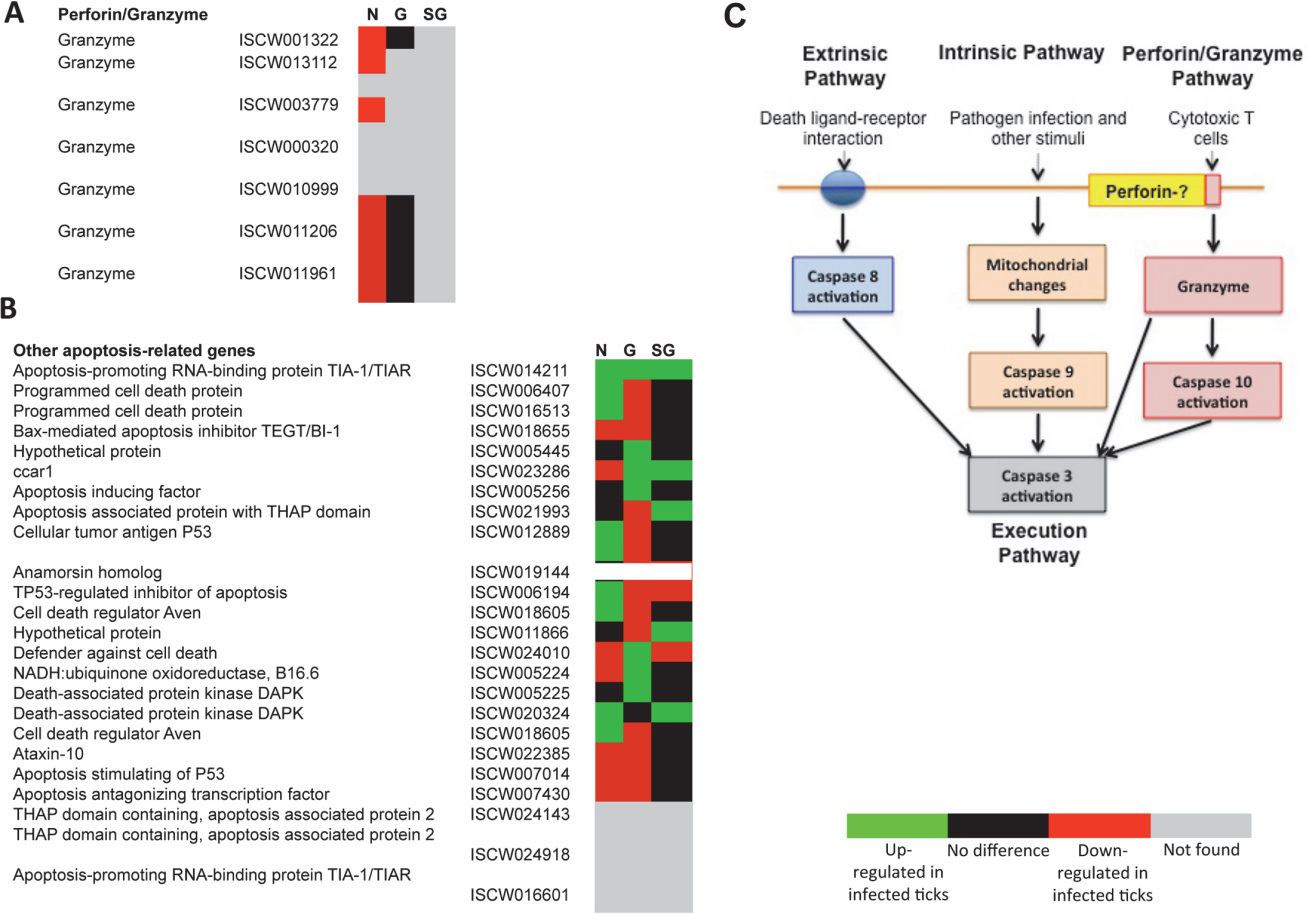
Annotation of Perforin/Granzyme and other apoptosis pathway genes and expression in response to *A*. *phagocytophilum* infection. All apoptosis pathways described in other organisms were identified in *I*. *scapularis*, except for the absence of the Perforin ortholog in the Perforin/Granzyme pathway. (A) Perforin/Granzyme pathway. (B) Other apoptosis-related genes. For annotation, gene identifiers were obtained from VectorBase (www.vectorbase.org) and compared to the corresponding pathways in *Drosophila melanogaster*, *Anopheles gambiae*, *Aedes aegypti* and *Homo sapiens*. Differential expression (P<0.05) is shown for tick nymphs (N) and adult midguts (G) and salivary glands (SG). (C) Schematic representation of predicted apoptosis pathways in *I*. *scapularis*.

The annotation of the putative apoptosis pathway genes in *I*. *scapularis* was based on sequence identity to genes reported in other organisms and used to characterize the tissue-specific response to *A*. *phagocytophilum* infection (Figs 3A-3C and 4A and 4B; S4 Table). All apoptosis pathways described in other organisms were identified in *I*. *scapularis* (Fig 4C). Each pathway requires specific triggering signals to begin an energy-dependent cascade of molecular events that activate the Caspase-dependent apoptosis execution pathway [32]. At least in humans, the Perforin/Granzyme pathway can only work in a Caspase-independent fashion through Granzyme A (Fig 4C) [32]. However, the ortholog for the Perforin gene was not identified in *I*. *scapularis* in these studies (Fig 4A and 4C).

**Fig 5:**
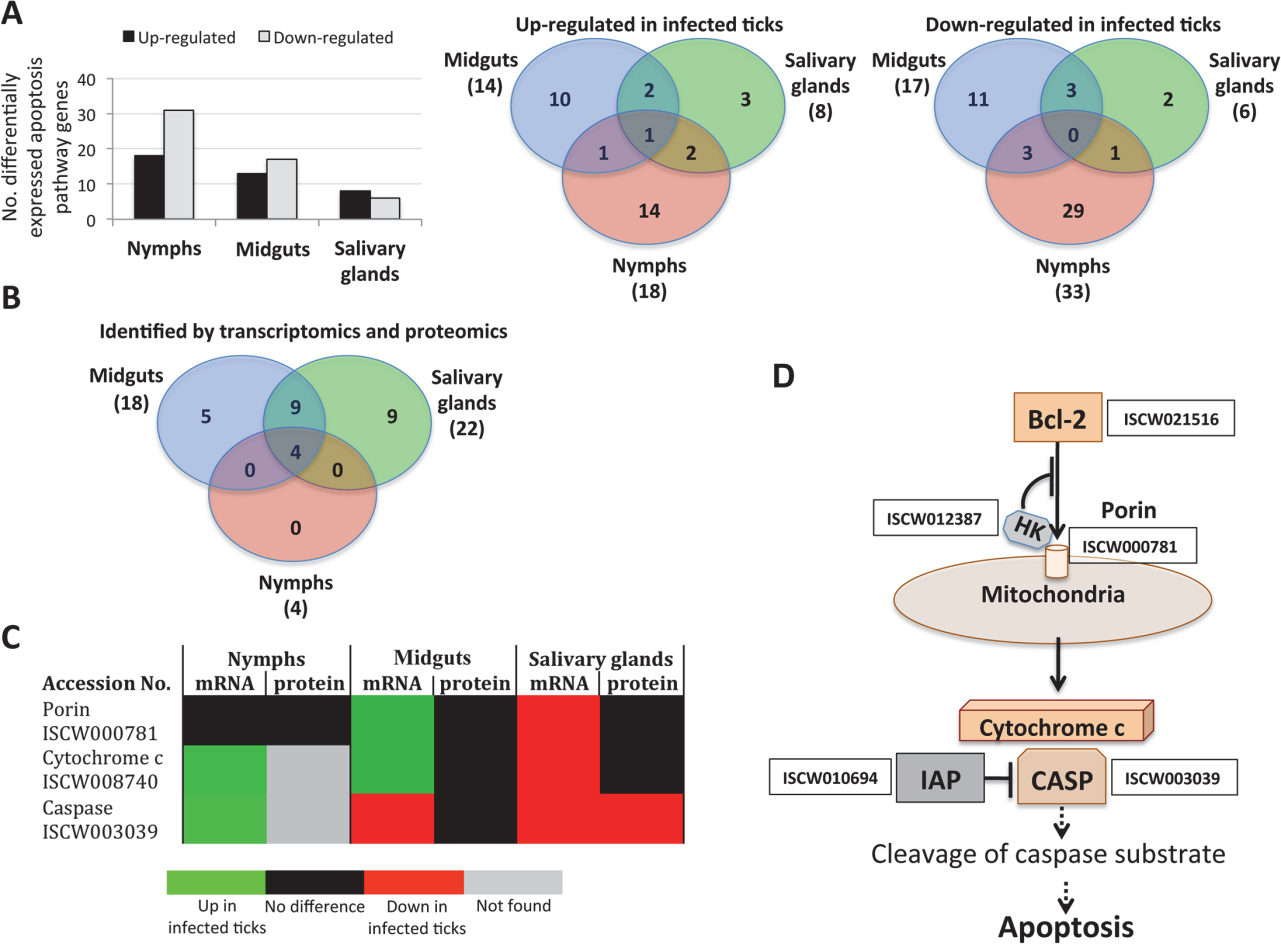
Characterization of apoptosis differentially expressed genes and represented proteins in response to *A*. *phagocytophilum* infection in *I*. *scapularis*. Apoptosis pathway gene and protein levels increased or decreased in a tissue-specific manner in response to pathogen infection, thus supporting the functional characterization of this biological pathway in infected in ticks. (A) Differentially expressed apoptosis genes in infected nymphs and adult female midguts and salivary glands. (B) Apoptosis genes/proteins identified by both transcriptomics and proteomics analyses in nymphs and adult female midguts and salivary glands. (C) Comparison between mRNA and proteins levels of selected intrinsic apoptosis pathway components in tick samples in response to pathogen infection. (D) Intrinsic apoptosis pathway genes selected for functional characterization by RNAi. The genes selected for RNAi are shown with the accession number next to them. Abbreviations: HK, Hexokinase; IAP, apoptosis inhibitor; CASP, Caspase.

Apoptosis pathway genes were differentially expressed in *I*. *scapularis* nymphs and adult female midguts and salivary glands with little overlapping between the different samples, thus providing additional evidences for the complexity of tissue-specific response to bacterial infection (Figs. 3A-3C, 4A, 4B and 5A; S4 Table). Four, 18 and 22 apoptosis pathway components were identified in both transcriptome and proteome in nymphs, adult female midguts and salivary glands, respectively (Fig 5B), and some of these molecules also showed differences between infected and uninfected samples at the protein level (S4 Table). These results suggested a role for apoptosis pathways during *A*. *phagocytophilum* infection of *I*. *scapularis*.

### *A*. *phagocytophilum* infection inhibits the intrinsic apoptosis pathway in tick salivary glands

Some of the intrinsic apoptosis pathway components demonstrated a clear pattern of gene/protein differential expression/representation among the various samples (Fig 5C). In nymphs and adult female midguts, a tendency was observed towards gene up-regulation without an effect on protein representation in response to infection. However, in adult female salivary glands genes were down-regulated in response to infection with Caspase protein under-represented in infected ticks.

One of the problems associated with gene/protein annotations based on sequence identity is that function may not be necessarily identical between organisms. Therefore, functional characterization is ultimately needed to support gene/protein annotation and predicted function. In ticks, RNA interference (RNAi) is the most widely used technique for functional analysis [33]. The intrinsic apoptosis pathway has been implicated in *A*. *phagocytophilum* infection of tick cells [16] and was therefore selected for functional analysis using RNAi (Fig 5D).

**Fig 6:**
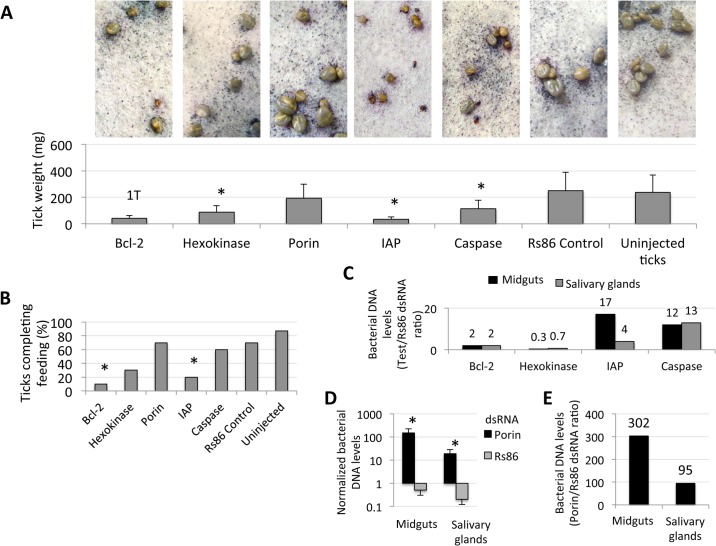
Intrinsic apoptosis gene knockdown phenotype in ticks injected with dsRNA. The knockdown of selected intrinsic apoptosis pathway genes had the greatest effects on tick feeding to repletion but, apparently, not on tick attachment. Porin silencing significantly increased tick colonization by *A*. *phagocytophilum* but did not affect tick feeding. Methods: Twenty adult female ticks were injected with gene-specific dsRNAs, the unrelated Rs86 dsRNA control or were left uninjected. After dsRNA injection, female ticks were allowed to feed on sheep inoculated intravenously with *A*. *phagocytophilum* (NY18 isolate). Female ticks were allowed to feed until full engorgement and tick weight and mortality were determined in individual female ticks collected after feeding. (A) Tick weight was compared between ticks injected with test genes dsRNA and Rs86 control dsRNA by Student's t-test with unequal variance (*P≤0.05). Panels show representative images at day 5 of tick feeding. (B) The number of ticks completing feeding was compared between ticks injected with test genes dsRNA and Rs86 control dsRNA by one-tailed Fisher's exact test (*P≤0.05). (C-E) *A*. *phagocytophilum* DNA levels were determined by *msp4* real-time PCR normalizing against tick 16S rDNA and shown as test dsRNA to Rs86 dsRNA ratio. Normalized Ct values were compared between ticks injected with test genes dsRNA and Rs86 control dsRNA by Student's t-test with unequal variance (*P<0.05) and were significantly different for Porin dsRNA-injected ticks only. Abbreviations: 1T, only one tick completed feeding; IAP, apoptosis inhibitor.

**Fig 7:**
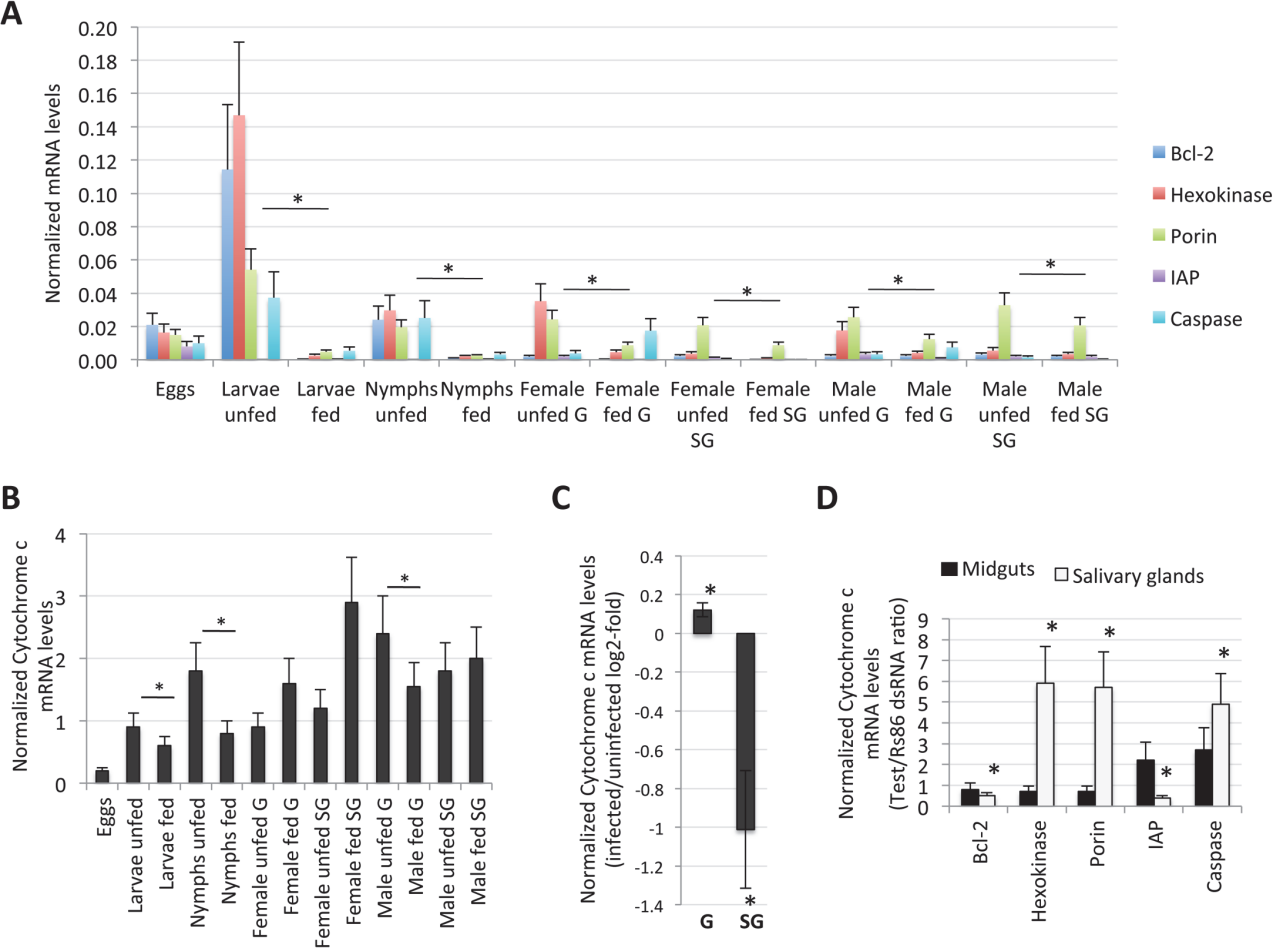
Expression of selected intrinsic apoptosis pathway genes. The analysis highlighted a complex mechanism by which tick cells respond to changes in the expression of intrinsic apoptosis pathway genes and suggested an effect of tick feeding on Porin expression that may also contributed to Porin down-regulation in infected adult female salivary glands. (A) Normalized Bcl-2, Hexokinase, Porin, IAP and Caspase mRNA levels (Ct values are shown as Ave+SD) in different tick developmental stages and tissues. (B) Normalized Cytochrome c mRNA levels are shown as Ave+SD in different tick developmental stages and tissues. The mRNA levels were characterized in tick eggs (three batches of approximately 500 eggs each), fed and unfed larvae (three pools of 50 larvae each), fed and unfed nymphs (three pools of 15 nymphs each), and fed and unfed males and females adults tick tissues (4 ticks each) by real-time RT-PCR normalizing against tick cyclophilin and ribosomal protein S4. Porin and Cytochrome c normalized Ct values were compared between fed and unfed ticks by Student's t-test with unequal variance (*P<0.05; N = 3–4). (C) Cytochrome c mRNA levels were determined in infected and uninfected female midguts (G) and salivary glands (SG), normalized against tick tick cyclophilin and ribosomal protein S4, represented as infected/uninfected Log2-fold ratio (Ave+SD) and normalized Ct values compared between infected and uninfected ticks by Student's t-test with unequal variance (*P<0.05; N = 10). (D) Cytochrome c mRNA levels were determined in midguts and salivary glands of ticks with gene knockdown, normalized against tick tick cyclophilin and ribosomal protein S4, represented as test/dsRNA ratio (Ave+SD) and normalized Ct values compared between ticks injected with test genes dsRNA and Rs86 control dsRNA by Student's t-test with unequal variance (*P<0.05). Abbreviation: IAP, apoptosis inhibitor.

The results revealed significant gene knockdown after dsRNA-mediated RNAi (Table 1). Gene knockdown for all selected intrinsic apoptosis pathway genes except Porin resulted in reduced tick weights (Fig 6A). These results differed from previous experiments in which Porin knockdown did result in reduced tick weigh [16]. One likely explanation for this discrepancy is the difference in the percent of gene expression silencing obtained in midguts, the most important organ in tick feeding, between both experiments (93% in [16] vs. 73% here (Table 1)), thus reinforcing that the role of Porin in tick feeding is marginal. The number of ticks that completed feeding was reduced in ticks injected with Bcl-2 and IAP dsRNAs (Fig 6B) and suggested a role for these genes during tick feeding. However, although a tendency was observed towards higher *A*. *phagocytophilum* DNA levels in ticks after RNAi for most of the selected intrinsic apoptosis pathway genes (Fig 6C), this effect was statistically significant for Porin only when compared to control dsRNA-injected ticks (Fig 6D and 6E). The results suggested that these genes do not have the same function reported in other organisms or the possible role of these genes on pathogen infection was not as relevant as that of Porin. Ayllón et al. [16] reported that *A*. *phagocytophilum* infection of tick cells results in down-regulation of mitochondrial Porin, thus providing a mechanism for subversion of host cell defenses to increase infection. This result was corroborated in these studies in which higher *A*. *phagocytophilum* DNA levels after Porin gene knockdown was found in both midguts and salivary glands (Fig 6D and 6E). Interestingly, among selected intrinsic apoptosis pathway genes, Porin was the only one consistently showing higher mRNA levels in unfed than in fed tick developmental stages and tissues (Fig 7A), suggesting an effect of tick feeding on Porin expression that may also contributed to Porin down-regulation in infected adult female salivary glands.

**Table 1:** Gene knockdown in tick midguts and salivary glands.

dsRNA	Ticks with gene knockdown (%)		Gene expression silencing (%) (Ave±SD; range)	
	Midguts	Salivary glands	Midguts	Salivary glands
Bcl-2	70	90	82±21*; 45–99	50±16*; 18–70
Hexokinase	70	90	89±6*; 82–98	80±30*; 13–100
Porin	80	100	73±21*; 28–86	70±28*; 21–99
IAP	100	80	100±0*; 99–100	60±15*; 37–71
Caspase	50	90	74±21*; 43–92	96±5*; 83–100

Twenty female ticks were injected with gene-specific dsRNA or Rs86 control dsRNA. Ten ticks per group were collected after 7 days of feeding and midguts and salivary glands dissected for RNA extraction to characterize gene knockdown by real-time RT-PCR with respect to Rs86 control. The mRNA levels were normalized against tick 16S rRNA and cyclophilin and compared between test dsRNA-treated ticks and controls treated with Rs86 dsRNA by Student's t-test with unequal variance (*P<0.05).

Tick feeding and infection with *A*. *phagocytophilum* may also affect Cytochrome c expression as part of the effect on the intrinsic apoptosis pathway (Fig 5C). Tick feeding resulted in variable Cytochrome c mRNA levels in different tissues and developmental stages with lower levels in fed larvae, nymphs and adult male midguts but not in female ticks and adult male salivary glands when compared to unfed ticks (Fig 7B). Infection with *A*. *phagocytophilum* resulted in up-regulation of Cytochrome c in adult female midguts but down-regulation in the salivary glands (Fig 7C), in agreement with Porin expression in response to infection (Fig 5C). The knockdown of intrinsic apoptosis pathway genes did not affect Cytochrome c mRNA levels in adult female tick midguts, but the effect in salivary glands suggested a complex mechanism by which tick cells respond to changes in the expression of these genes (Fig 7D). Taken together, these results showed a complex effect of tick feeding and *A*. *phagocytophilum* infection on Cytochrome c mRNA levels.

**Fig 8:**
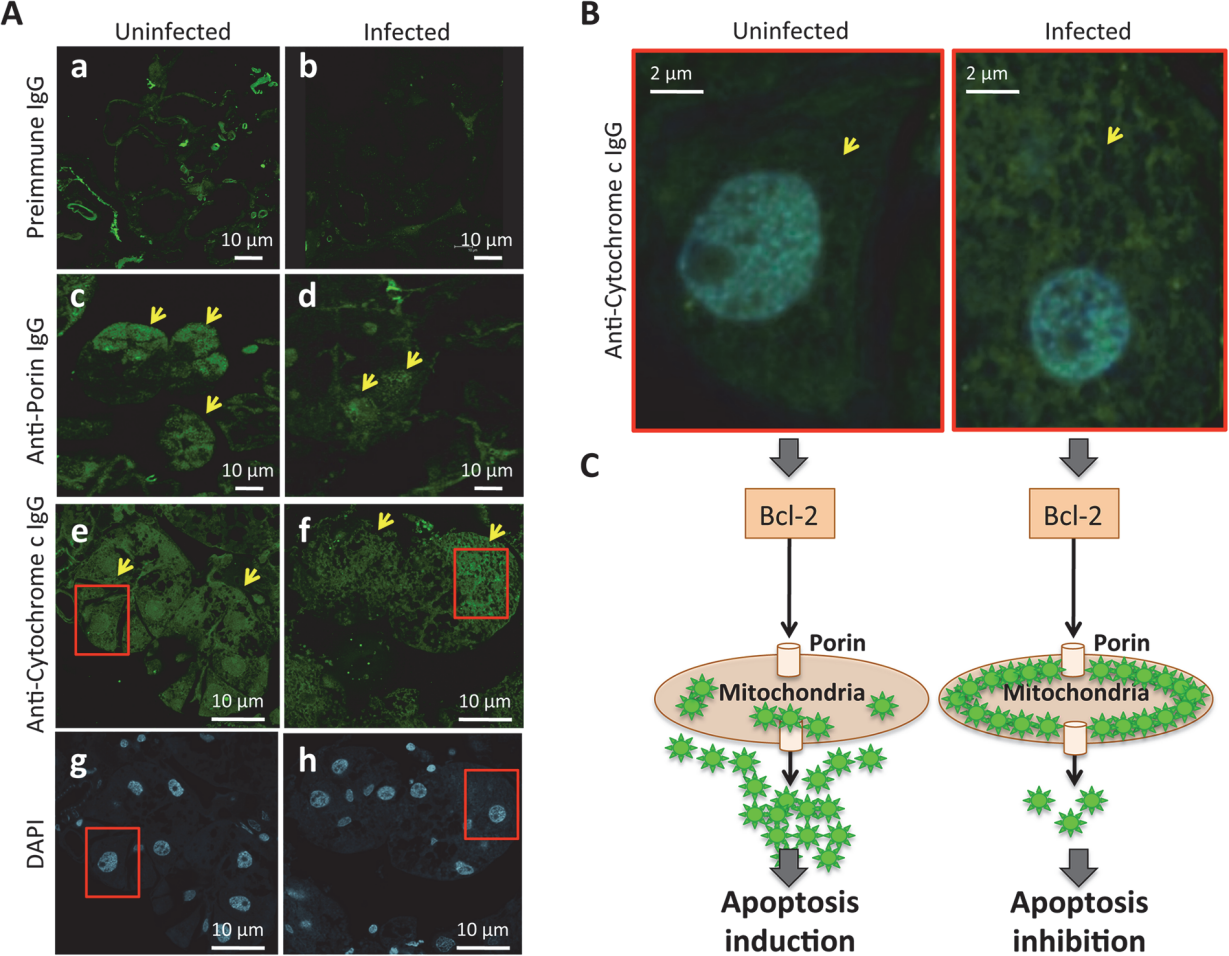
Immunohistochemical localization of tick Porin and Cytochrome c. The results showed that Porin levels were lower in infected than in uninfected tick salivary glands but demonstrated that differences in Cytochrome c levels were not at the protein level but in the localization of Cytochrome c, which was distributed in the cell cytoplasm of uninfected tick salivary glands but was mainly localized within organelles that probably corresponded to mitochondria in *A*. *phagocytophilum*-infected tick salivary glands. (A) Representative images of immunofluorescence analysis of uninfected and *A*. *phagocytophilum*-infected adult female tick salivary glands. Tick tissues were stained with rabbit anti-tick proteins antibodies (green, FITC). Arrows show positive staining in response to anti-Porin and anti-Cytochrome c IgG not present after incubation with preimmune IgG. (a-b) preimmune control serum-treated cells, which gave similar results between uninfected and infected ticks. (c-d) uninfected and infected cells stained with anti-Porin antibodies. (e-f) uninfected and infected cells stained with anti-Cytochrome c antibodies. (g-h) uninfected and infected cells stained with DAPI. Bars, 10 μm. (B) Sections in the red squares in e-h were magnified 5X and superimposed to illustrate the localization of the Cytochrome c in the cytoplasm of uninfected and infected adult female tick salivary glands. Arrows show positive staining. (C) Model of the Porin-mediated inhibition of Cytochrome c release as the anti-apoptotic mechanism to facilitate bacterial infection of tick salivary glands.

Although Porin and Cytochrome c expression was down-regulated in infected tick salivary glands, differences in protein levels between uninfected and *A*. *phagocytophilum*-infected tick salivary glands were not found (Fig 5C). These results were corroborated by immunocytochemistry (Fig 8A), demonstrating that differences between infected and uninfected tick salivary glands were not at the protein level but in the localization of Cytochrome c (Fig 8B). While Cytochrome c was distributed in the cell cytoplasm of uninfected tick salivary glands, in *A*. *phagocytophilum*-infected tick salivary glands Cytochrome c was mainly localized within organelles that probably correspond to mitochondria (Fig 8C). Although the mechanism(s) regulating mitochondrial permeability and the release of Cytochrome c during apoptosis are not fully understood, Bcl-2 may acts through the voltage-dependent anion channel or Porin, which in turn may play a role in regulating Cytochrome c release [34]. Taken together, these results demonstrated that *A*. *phagocytophilum* infection results in down-regulation of Porin expression in tick salivary gland but not midgut cells, which did not translate in different protein levels but resulted in the inhibition of Cytochrome c release as the anti-apoptotic mechanism to facilitate bacterial infection (Fig 8C).

### The extrinsic apoptosis pathway is activated in tick salivary glands in response to *A*. *phagocytophilum* infection

**Fig 9:**
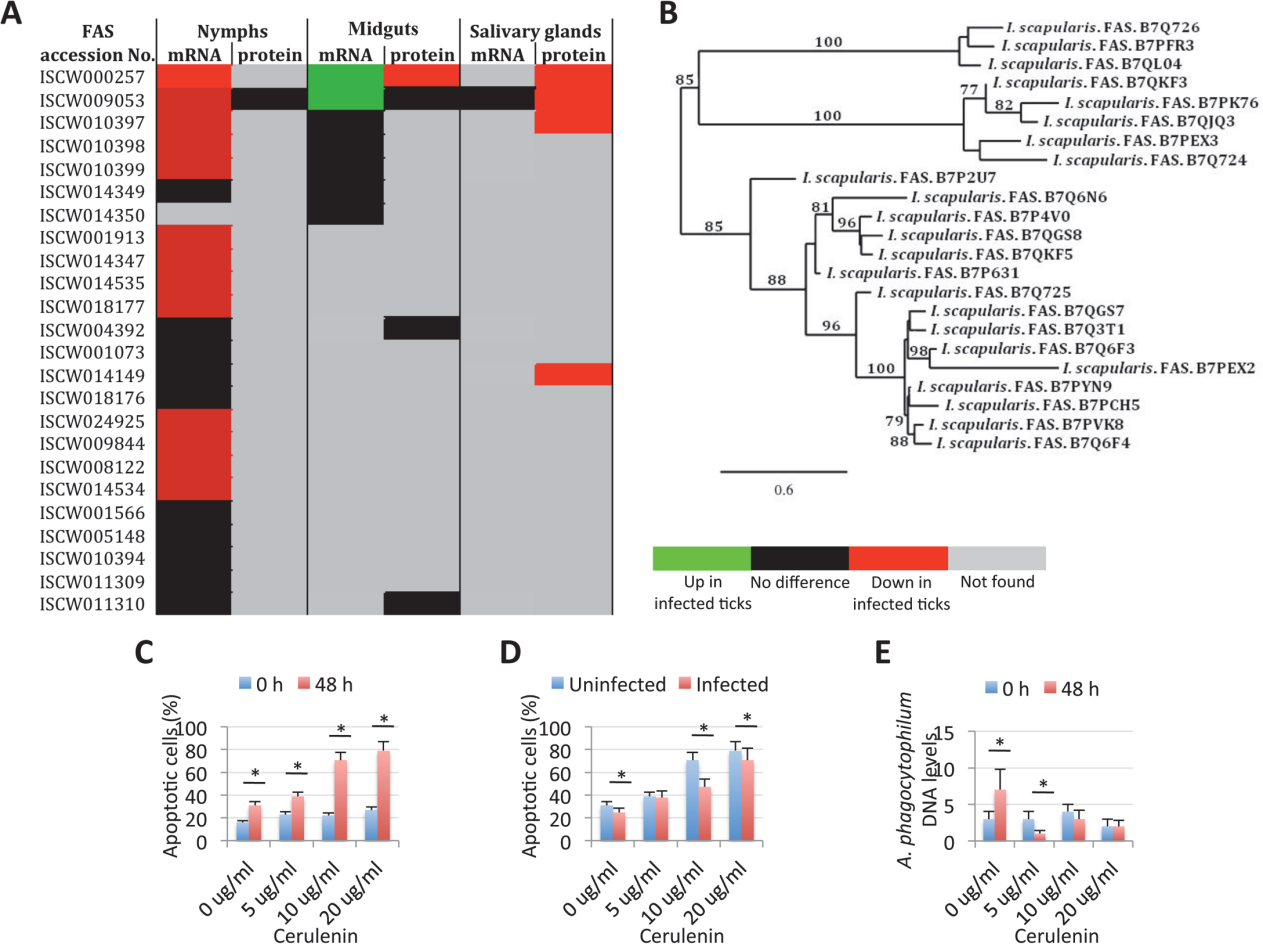
Role of tick Fatty acid synthase (FAS) in response to *A*. *phagocytophilum* infection. The expression of FAS genes was down-regulated in tick salivary glands, suggesting a response to *A*. *phagocytophilum* infection by promoting apoptosis to limit bacterial infection through induction of the extrinsic apoptosis pathway. Cerulenin had an effect on cultured tick cells by promoting apoptosis through FAS inhibition and results corroborated the effect of FAS inhibition on reducing *A*. *phagocytophilum* infection of tick salivary glands by activating the extrinsic apoptosis pathway in response to bacterial infection. The JAK/STAT pathway genes were down-regulated in nymphs, up-regulated in adult female midguts and not affected by bacterial infection in adult female salivary glands, suggesting that *A*. *phagocytophilum* infection decreased immunity in nymphs while inhibited cell apoptosis in midgut cells to facilitate and establish infection. (A) Comparison of FAS mRNA and protein levels in tick samples in response to pathogen infection. (B) Phylogenetic analysis of *I*. *scapularis* FAS amino acid sequences. The tree was constructed using the maximum likelihood method. Bootstrap values are represented as percent on internal branches (1000 replicates). Only Bootstrap values higher than 70 are shown. The GenBank accession numbers of the sequences used in the phylogenetic analysis are shown. (C) The percent of apoptotic cells was determined by flow cytometry in uninfected ISE6 tick cells treated for 48 h with different concentrations of the FAS inhibitor, Cerulenin. Results were represented as Ave+SD and compared between cells analyzed at 0 and 48 h of Cerulenin treatment by Student's t-test with unequal variance (*P<0.05; N = 3). (D) The percent of apoptotic cells was determined by flow cytometry in *A*. *phagocytophilum*-infected ISE6 tick cells treated for 48 h with different concentrations of the FAS inhibitor, Cerulenin. Results were represented as Ave+SD and compared between uninfected and infected cells by Student's t-test with unequal variance (*P<0.05; N = 3). (E) *A*. *phagocytophilum* DNA levels were determined in infected ISE6 tick cells treated for 48 h with different concentrations of the FAS inhibitor, Cerulenin by *msp4* real-time PCR normalizing against tick 16S rDNA. Results are shown as Ave+SD normalized Ct values and were compared between cells analyzed at 0 and 48 h of Cerulenin treatment and bacterial infection by Student's t-test with unequal variance (*P<0.05; N = 3).

The extrinsic apoptosis pathway is composed of Death ligand/receptor interactions such as Fatty acid synthase (FAS) ligand (FasL)/receptor that activate apoptosis (Fig 4C). A putative FasL-coding gene was not identified in the *I*. *scapularis* genome sequence, but the identification of the Fas apoptotic inhibitory molecule and Death receptors suggested that still uncharacterized FasL-like ligands may be present in ticks. Two genes were annotated as coding for Death receptors but except for down-regulation in nymphs for one of them, their expression did not change in response to pathogen infection and were not identified in the tick proteome (S4 Table). FAS is a central enzyme in *de novo* lipogenesis [35] but the inhibition of FAS causes apoptosis [36–39]. Interestingly, 24 genes were annotated as FAS-coding genes (Fig 3B). In general, most of the putative FAS proteins were not identified in the tick proteome, suggesting low protein levels or problems with the annotation of these genes (Fig 9A). Nevertheless, 6 of the putative FAS genes were corroborated at the protein level (Fig 9A). The analysis was then focused on the changes in FAS mRNA and protein levels in response to *A*. *phagocytophilum* infection that revealed different patterns in tick nymphs and adult female midguts and salivary glands (Fig 9A). Thirteen FAS genes were down-regulated in tick nymphs while two FAS genes were up-regulated in adult female midguts in response to infection. In adult female salivary glands, FAS gene expression could not be assessed but *A*. *phagocytophilum* infection resulted in 4 under-represented FAS proteins (Fig 9A).

Two different mechanisms mediated by the extrinsic [36, 37] and intrinsic [38, 39] pathways have been proposed to explain the apoptosis induced by the inhibition of FAS. However, the activation of the intrinsic apoptosis pathway is associated with mitochondrial oxidative stress and respiratory chain impairment, independent of FAS inhibition [39], thus suggesting that tick salivary glands may be responding to *A*. *phagocytophilum* infection by promoting apoptosis to limit bacterial infection through induction of the extrinsic apoptosis pathway. In this way, activation of the extrinsic apoptosis pathway in infected salivary glands may serve to counteract, at least in part, bacterial inhibition of the intrinsic apoptosis pathway. The activation of the extrinsic apoptosis pathway after FAS inhibition may be mediated by different mechanisms including possible interactions between FAS and FasL [36, 37, 40].

Phylogenetic analysis of putative *I*. *scapularis* FAS proteins suggested functional redundancy (Fig 9B), thus encouraging the use of FAS inhibitors and not RNAi for the functional characterization of these molecules during *A*. *phagocytophilum* infection of tick cells. Despite the increase in the number of apoptotic uninfected tick cells in culture, a 2 to 3 fold increase in the percent of apoptotic cells after 48 h of treatment with 5, 10 or 20 μg/ml of the FAS inhibitor Cerulenin was observed (Fig 9C). These results suggested that, as in other organisms [41], Cerulenin had an effect on cultured tick cells by promoting apoptosis through FAS inhibition. After 48 h of *A*. *phagocytophilum* infection of tick cells, the percent of apoptotic cells decreased in the presence of 0, 10 and 20 μg/ml of Cerulenin (Fig 9D), probably reflecting the effect of bacterial infection on the inhibition of the intrinsic apoptosis pathway. However, as expected for the Cerulenin induction of the extrinsic apoptosis pathway, *A*. *phagocytophilum* DNA levels decreased after 48 h of treatment as compared with infection levels in the absence of Cerulenin (Fig 9E). These results corroborated the effect of FAS inhibition on reducing *A*. *phagocytophilum* infection of tick salivary glands by activating the extrinsic apoptosis pathway in response to bacterial infection.

### The tick JAK/STAT pathway plays a role during *A*. *phagocytophilum* infection

**Fig 10:**
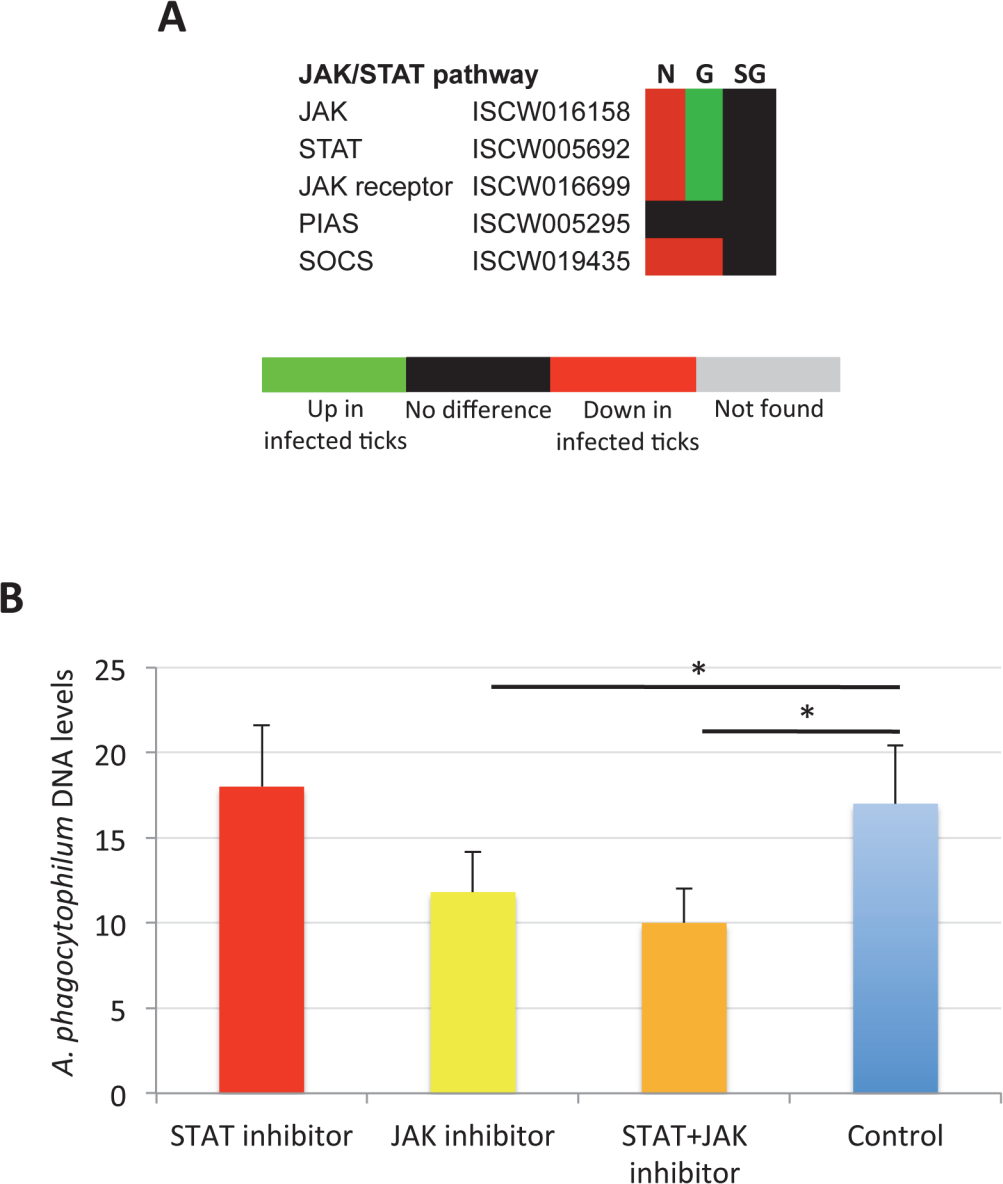
Role of tick JAK/STAT pathway in response to *A*. *phagocytophilum* infection. The JAK/STAT pathway genes were down-regulated in nymphs, up-regulated in adult female midguts and not affected by bacterial infection in adult female salivary glands, suggesting as corroborated by the treatment of infected tick cells with JAK-STAT inhibitors, that *A*. *phagocytophilum* infection decreases immunity in nymphs while inhibiting cell apoptosis in midgut cells to facilitate and establish infection. (A) Annotation of JAK/STAT pathway genes and expression in response to *A*. *phagocytophilum* infection. For annotation, gene identifiers were obtained from VectorBase (www.vectorbase.org) and compared to the corresponding pathways in *Drosophila melanogaster*, *Anopheles gambiae*, *Aedes aegypti* and *Homo sapiens*. Differential expression (P<0.05) is shown for tick nymphs (N) and adult midguts (G) and salivary glands (SG). (B) *A*. *phagocytophilum* DNA levels were determined in infected ISE6 tick cells untreated (Control) or treated for 48 h with STAT (9.2 μM), JAK (400 nM), and STAT (9.2 μM) + JAK (400 nM) inhibitors. Bacterial DNA levels were determined by *msp4* real-time PCR normalizing against tick 16S rDNA. Results are shown as Ave+SD normalized Ct values and were compared between untreated and treated cells by Student's t-test with unequal variance (*P<0.05; N = 4).

The Janus kinase/signal transducers and activators of transcription (JAK/STAT) pathway has been implicated in apoptosis signaling in vertebrate hosts infected with *A*. *phagocytophilum* [13], but has not been previously characterized in infected ticks. The JAK/STAT pathway genes were down-regulated in nymphs, up-regulated in adult female midguts and not affected by bacterial infection in adult female salivary glands (Fig 10A). In vertebrate hosts, *A*. *phagocytophilum* infection activates the JAK/STAT pathway to inhibit neutrophil apoptosis while mycobacteria and Brucellae inhibit this pathway to overcome host adaptive immunity [13]. The results in ticks suggested that similar mechanisms might occur during *A*. *phagocytophilum* infection by decreasing immunity in nymphs while inhibiting cell apoptosis in midgut cells to facilitate and establish infection. However, none of the JAK/STAT pathway components were identified in the tick proteome (S4 Table), thus precluding from comparing mRNA and protein levels in infected tick samples.

To verify the possible role of the tick JAK/STAT pathway during *A*. *phagocytophilum* infection, a preliminary experiment was conducted treating infected tick cells with JAK and/or STAT inhibitors (Fig 10B). The results showed that while the STAT inhibitor did not affect bacterial infection, treatment with the JAK inhibitor and the combination of STAT and JAK inhibitors did result in the reduction of *A*. *phagocytophilum* DNA levels when compared to control cells incubated with growth medium alone. These results supported a role for the tick JAK/STAT pathway during *A*. *phagocytophilum* infection.

### Validation of RNAseq and proteomics data

The validation of RNAseq and proteomics data is important in order to provide additional support for the results obtained in these studies. However, although real-time RT-PCR is easy to perform to validate RNAseq data, few antibodies are available against tick proteins for validation of proteomics data. Herein, 16 genes were selected to validate RNAseq results by real-time RT-PCR (S6A Fig). Analysis of mRNA levels by real-time RT-PCR in individual samples from infected and uninfected tick nymphs, adult female midguts and salivary glands corroborated RNAseq results by demonstrating that gene up- or down-regulation was similar between RNAseq and RT-PCR analyses for most samples (S6B Fig). The minor differences observed between the results of both analyses could be attributed to intrinsic variation in gene expression and the fact that approximately 85% of the ticks used for RNAseq were infected [42] while for RT-PCR all ticks were confirmed uninfected or infected with *A*. *phagocytophilum* before analysis. Nevertheless, a positive correlation was obtained for absolute differential expression values between RNAseq and RT-PCR (S6C Fig). For the validation of proteomics data only nymph proteins were available after completion of the studies and two antibodies against intrinsic apoptosis pathway proteins, Porin and Cytochrome c, were used for Western blot analysis (S6D Fig) and immunofluorescence (Fig 8A). The results corroborated proteomics results in adult female tick salivary glands and nymphs and showed that although Cytochrome c was not identified by proteomics in nymphs (Fig 5C), Western blot results did not show any difference between infected and uninfected ticks (S6D Fig).

### Conclusions

The experimental approach used in this study using systems biology showed a dramatic and complex tissue-specific response to *A*. *phagocytophilum* in the tick vector, *I*. *scapularis*. The results demonstrated that tick tissue-specific response to infection reflected pathogen developmental cycle and the impact of *A*. *phagocytophilum* infection on tick apoptosis pathways in a tissue-specific manner.

**Fig 11:**
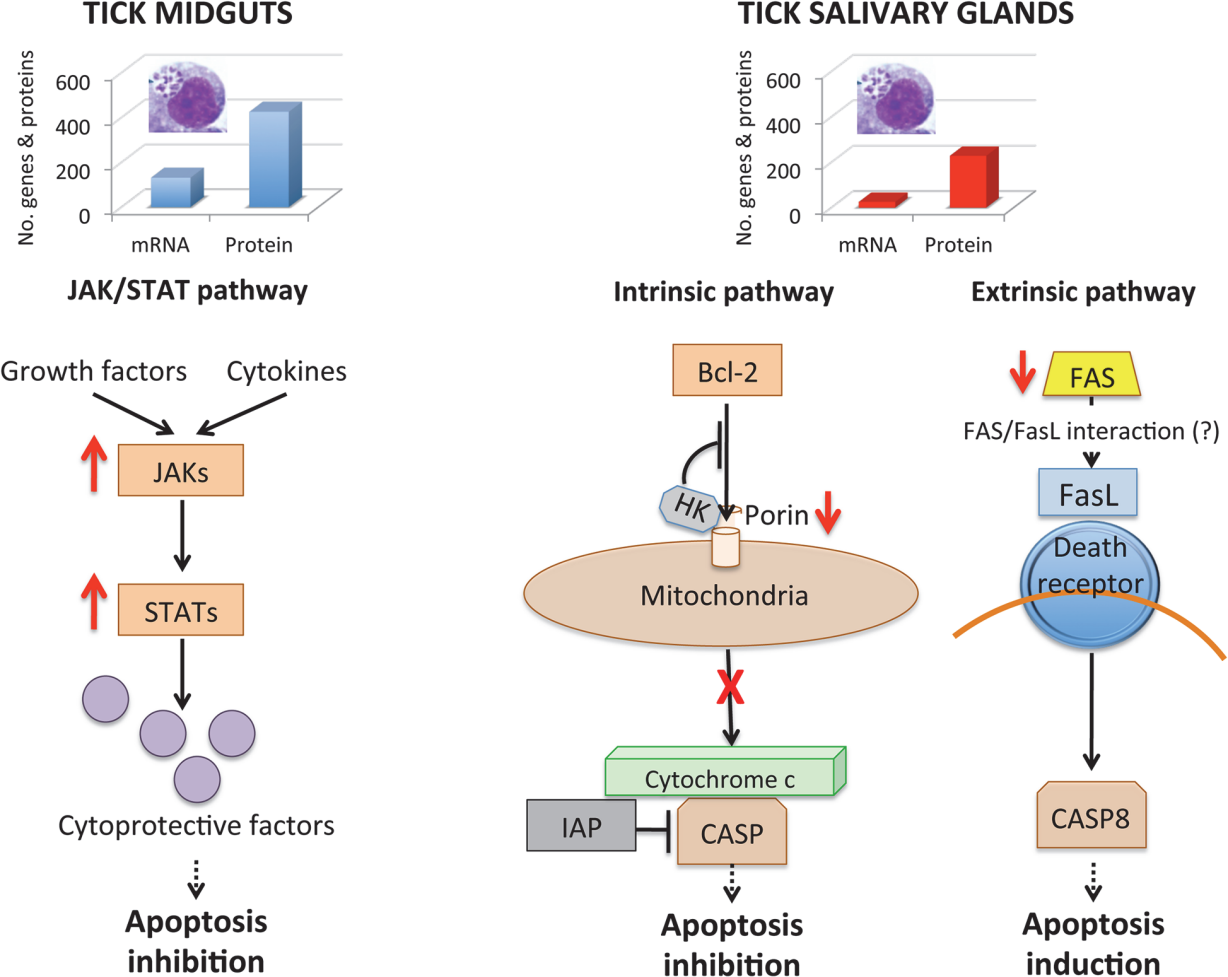
Effect of *A*. *phagocytophilum* infection on adult female midguts and salivary glands. The number of highly differentially expressed genes and represented proteins was higher in tick midguts than in salivary glands, suggesting a higher impact of bacterial infection in midgut cells probably associated with *A*. *phagocytophilum* developmental cycle. In tick midguts, *A*. *phagocytophilum* inhibited cell apoptosis to facilitate and establish infection through up-regulation of the JAK/STAT pathway genes. In tick salivary glands, down-regulation of Porin resulted in the inhibition of the Cytochrome c release that inhibited the mitochondrially-induced intrinsic apoptosis pathway to facilitate bacterial infection. This effect was contrasted in part by the induction of the extrinsic apoptosis pathway through the inhibition of FAS proteins.

All apoptosis pathways described in other organisms were identified in *I*. *scapularis*, except for the absence of the Perforin ortholog in the Perforin/Granzyme pathway, and tissue-specific differences were found in the response to *A*. *phagocytophilum* infection. Although an ortholog for FasL was not identified in *I*. *scapularis*, other Death ligand/receptor interactions may activate the extrinsic apoptosis pathway. Functional characterization using RNAi demonstrated that Porin silencing significantly increased tick colonization by *A*. *phagocytophilum* but did not affect tick feeding, thus illustrating how bacterial inhibition of Porin expression increases tick vector capacity for this pathogen. In tick nymphs, the results suggested a possible effect of bacterial infection on the inhibition of tick immune response but further experiments are required to address this hypothesis. In tick midgut cells, the results suggested that *A*. *phagocytophilum* infection inhibited cell apoptosis to facilitate and establish infection through up-regulation of the JAK/STAT pathway genes. Bacterial infection inhibited the intrinsic apoptosis pathway in tick salivary glands but not in midguts by down-regulating Porin expression that resulted in the inhibition of Cytochrome c release as the anti-apoptotic mechanism to facilitate bacterial infection. However, tick salivary glands may be responding to *A*. *phagocytophilum* by promoting apoptosis to limit bacterial infection through induction of the extrinsic apoptosis pathway. In summary, the results suggested that *A*. *phagocytophilum* uses different mechanisms to establish infection in *I*. *scapularis* nymphs and adult female midguts and salivary glands (Fig 11), supporting the observation that the pathogen uses similar strategies to establish infection in both vertebrate and invertebrate hosts [16]. *A*. *phagocytophilum* has a type IV secretion system that translocates effector molecules to host cells to exert their activity on transcription and apoptosis and favor bacterial infection [27, 31]. These effectors have not been fully characterized but may be responsible for some of the changes shown here to occur in tick transcriptome and proteome in response to bacterial infection. These dynamic changes in response to *A*. *phagocytophilum* in *I*. *scapularis* tissue-specific transcriptome and proteome demonstrated the complexity of the tick response to infection and will contribute to characterize gene regulation in ticks.

## Materials and Methods

### Ethics statement

Animals were housed and experiments conducted with the approval and supervision of the OSU Institutional Animal Care and Use Committee (Animal Care and Use Protocol, ACUP No. VM1026).

### Ticks and sample preparation

*I*. *scapularis *ticks were obtained from the laboratory colony maintained at the Oklahoma State University Tick Rearing Facility. Larvae and nymphs were fed on rabbits and adults were fed on sheep. Off-host ticks were maintained in a 12 hr light: 12 hr dark photoperiod at 22–25°C and 95% relative humidity. Nymphs and adult female *I*. *scapularis* were infected with *A*. *phagocytophilum* by feeding on a sheep inoculated intravenously with approximately 1x10^7^ *A*. *phagocytophilum* (NY18 isolate)-infected HL-60 cells (90–100% infected cells) [42]. In this model, over 85% of ticks become infected with *A*. *phagocytophilum* in nymphs, midguts and salivary glands [42]. Ticks (200 nymphs and 100 female adults) were removed from the sheep 7 days after infestation, held in the humidity chamber for 4 days and dissected for DNA, RNA and protein extraction from whole internal tissues (nymphs) or midguts and salivary glands (adult females). Adult midguts and salivary glands were washed in PBS after collection to remove hemolymphs-related cells. Uninfected ticks were prepared in a similar way but feeding on an uninfected sheep. Two independent samples were collected and processed for each tick developmental stage and tissue. Total RNA, DNA and proteins were extracted from uninfected and *A*. *phagocytophilum*-infected nymph, midgut and salivary gland samples using the AllPrep DNA/RNA/Protein Mini Kit (Qiagen, Valencia, CA, USA). Ten individual nymphs and female ticks were dissected and samples collected to characterize *A*. *phagocytophilum* infection and the mRNA levels of genes selected after RNA sequencing (RNAseq).

### RNA sequencing and analysis

Total RNA quality was evaluated using the Agilent 2100 Bioanalyzer RNA Nano Chip (Agilent Technologies, Santa Clara, CA, USA). For RNAseq sample preparation, the TruSeq Stranded mRNA Sample Prep Kit (Illumina, San Diego, CA, USA) was used according to the manufacturer's protocol. Briefly, 10 μg of each total RNA sample was used for polyA mRNA selection using streptavidin-coated magnetic beads, followed by thermal mRNA fragmentation. The fragmented mRNA was subjected to cDNA synthesis using the SuperScript II reverse transcriptase (Life Technologies, Grand Island, NY, USA) and random primers. The cDNA was further converted into double stranded cDNA and, after an end repair process, was finally ligated to Illumina paired end (PE) adaptors. Size selection was performed using a 2% agarose gel, generating cDNA libraries ranging in size from 200–500 bp. Finally, the libraries were enriched using 15 cycles of PCR and purified by the QIAquick PCR purification kit (Qiagen, Valencia, CA, USA). The enriched libraries were diluted with elution buffer (Qiagen) to a final concentration of 10 nM. Each library was run at a concentration of 7 pM on one Illumina Hiseq 2000 lane using 100 bp sequencing (CD BioSciences, Shirley, NY, USA). In the case of paired-end reads, distinct adaptors from Illumina were ligated to each end with PCR primers that allowed reading of each end as separate runs. The sequencing reaction was run for 100 cycles (tagging, imaging, and cleavage of one terminal base at a time), and four images of each tile on the chip were taken in different wavelengths for exciting each base-specific fluorophore. For paired-end reads, data were collected as two sets of matched 100-bp reads. Reads for each of the indexed samples were then separated using a custom Perl script. Image analysis and base calling were done using the Illumina GA Pipeline software.

TopHat [43] was used to align the reads to the *I*. *scapularis* (assembly JCVI_ISG_i3_1.0; http://www.ncbi.nlm.nih.gov/nuccore/NZ_ABJB000000000) reference genome. TopHat incorporates the Bowtie algorithm to perform the alignment [44]. TopHat initially removes a portion of reads based on quality information accompanying each read, then maps reads to the reference genome. TopHat allows multiple alignments per read (up to 40 by default) and a maximum of 2 mismatches when mapping reads to the reference genome. The mapping results were then used to identify “islands” of expression, which can be interpreted as potential exons. TopHat builds a database of potential splice junctions and confirms these by comparing the previously unmapped reads against the database of putative junctions. Default parameters for TopHat were used. Raw counts per gene were estimated by the Python script HTSeq count [http://www-huber.embl.de/users/anders/HTSeq/] using the reference genome. The raw counts per gene were used by DEGseq [45] to estimate differential expression at P<0.05.

### Proteomics data collection and analysis

Proteins were digested using the filter aided sample preparation (FASP) protocol [46]. The FASP method allows processing total SDS lysates of essentially any class of protein from biological samples of any origin, thus solving the long-standing problem of efficient and unbiased solubilization of all cellular proteins irrespective of their subcellular localization and molecular weight. Briefly, samples were dissolved in 50 mM Tris-HCl pH8.5, 4% SDS and 50 mM DTT, boiled for 10 min and centrifuged. Protein concentration in the supernatant was measured by the Direct Detect system (Millipore, Billerica, MA, USA). About 150 μg of protein were diluted in 8 M urea in 0.1 M Tris-HCl (pH 8.5) (UA), and loaded onto 30 kDa centrifugal filter devices (FASP Protein Digestion Kit, Expedeon, TN, USA). With this method, the sample is solubilized in 4% SDS, then retained and concentrated into microliter volumes in an ultrafiltration device. The filter unit then acts as a ‘proteomic reactor’ for detergent removal, buffer exchange, chemical modification and protein digestion. Notably, during peptide elution, the filter retains high-molecular-weight substances that would otherwise interfere with subsequent peptide separation [46]. The denaturation buffer was replaced by washing three times with UA. Proteins were later alkylated using 50 mM iodoacetamide in UA for 20 min in the dark, and the excess of alkylation reagents were eliminated by washing three times with UA and three additional times with 50 mM ammonium bicarbonate. Proteins were digested overnight at 37°C with modified trypsin (Promega, Madison, WI, USA) in 50 mM ammonium bicarbonate at 40:1 protein:trypsin (w/w) ratio. The resulting peptides were eluted by centrifugation with 50 mM ammonium bicarbonate (twice) and 0.5 M sodium chloride. Trifluoroacetic acid (TFA) was added to a final concentration of 1% and the peptides were finally desalted onto C18 Oasis-HLB cartridges and dried-down for further analysis. For stable isobaric labeling, the resulting tryptic peptides were dissolved in Triethylammonium bicarbonate (TEAB) buffer and labeled using the 4-plex iTRAQ Reagents Multiplex Kit (Applied Biosystems, Foster City, CA, USA) according to manufacturer's protocol. Briefly, each peptide solution was independently labeled at room temperature for 1 h with one iTRAQ reagent vial (mass tag 114, 115, 116 or 117) previously reconstituted with 70 μl of ethanol. Reaction was stopped after incubation at room temperature for 1 h with diluted TFA, and peptides were combined. Samples were evaporated in a Speed Vac, desalted onto C18 Oasis-HLB cartridges and dried-down for further analysis as previously described. Labeled peptides were loaded into the LC-MS/MS system for on-line desalting onto C18 cartridges and analyzing by LC-MS/MS using a C-18 reversed phase nano-column (75 μm I.D. x 50 cm, 3 μm particle size, Acclaim PepMap 100 C18; Thermo Fisher Scientific, Waltham, MA, USA) in a continuous acetonitrile gradient consisting of 0–30% B in 145 min, 30–43% A in 5 min and 43–90% B in 1 min (A = 0.5% formic acid; B = 90% acetonitrile, 0.5% formic acid). A flow rate of ca. 300 nl/min was used to elute peptides from the reverse phase nano-column to an emitter nanospray needle for real time ionization and peptide fragmentation on orbital ion trap mass spectrometers (both Orbitrap Elite and QExactive mass spectrometers, Thermo Fisher Scientific). For increasing proteome coverage, iTRAQ-labeled samples were also fractionated by cation exchange chromatography (Oasis HLB-MCX columns) into six fractions, which were desalted and analyzed by using the same system and conditions described before. For peptide identification, all spectra were analyzed with Proteome Discoverer (version 1.4.0.29, Thermo Fisher Scientific) using a Uniprot database containing all sequences from Ixodida (May 17, 2013). For database searching, parameters were selected as follows: trypsin digestion with 2 maximum missed cleavage sites, precursor and fragment mass tolerances for the Elite of 600 ppm and 1200 mmu, respectively, or 2 Da and 0.02 Da, respectively for the QExactive, carbamidomethyl cysteine as fixed modification and methionine oxidation as dynamic modifications. For iTRAQ labeled peptides, N-terminal and Lys iTRAQ modification was added as a fixed modification. Peptide identification was validated using the probability ratio method [47] and false discovery rate (FDR) was calculated using inverted databases and the refined method [48] with an additional filtering for precursor mass tolerance of 12 ppm. Only peptides with a confidence of at least 95% were used to quantify the relative abundance of each peptide determined as described previously [49].

Protein quantification from reporter ion intensities and statistical analysis of quantitative data were performed as described previously using QuiXoT [50, 51]. For iTRAQ data, only the intensity of the reporter ions within 0.4 Da windows around the theoretical values was considered for quantification. Reporter intensities were corrected for isotopic contaminants by taking into consideration the information provided by the manufacturer. The intensity of the reporter peaks was used to calculate the fitting weight of each spectrum in the statistical model as described previously [51]. Outliers at the scan and peptide levels and significant protein-abundance changes were detected from the z values (the standardized variable used by the model that expresses the quantitative values in units of standard deviation) by using a false discovery rate (FDR) threshold of 5% as described previously [51]. Results are the mean of two replicates.

### Gene and protein ontology analysis

The gene and proteins ontology (GO) analysis for Biological Process (BP) and Molecular Function (MF) was done using the STRAP software (Software for Researching Annotations of Proteins; [http://www.bumc.bu.edu/cardiovascularproteomics/cpctools/strap/] developed at the Cardiovascular Proteomics Center of Boston University School of Medicine (Boston, MA, USA) [52]. For annotation of selected pathways, gene identifiers were obtained from VectorBase (www.vectorbase.org) and compared to the corresponding pathways in *D*. *melanogaster*, *Anopheles gambiae*, *Aedes aegypti* and *Homo sapiens*. Regression analysis of biological processes in infected tick nymphs, adult female midguts and salivary glands was conducted using Excel normalizing against the total number of differentially expressed genes and represented proteins and excluding transcripts and proteins without known assignations.

### RNA interference (RNAi) for gene knockdown in ticks

For RNAi, oligonucleotide primers containing T7 promoters (S5 Table) were used for *in vitro* transcription and synthesis of dsRNA as described previously [16], using the Access RT-PCR system (Promega) and the Megascript RNAi kit (Ambion, Austin, TX, USA). The unrelated gene Rs86 dsRNA was synthesized using the same methods described previously and used as negative control [16]. The dsRNA was purified and quantified by spectrophotometry. Unfed adult ticks (N = 20 females per group) were injected with approximately 0.5 μl dsRNA (5x10^10^-5x10^11^ molecules/μl) in the lower right quadrant of the ventral surface of the exoskeleton of ticks [53]. The injections were done using a 10-μl syringe with a 1-inch, 33 gauge needle (Hamilton, Bonaduz, Switzerland). Control ticks were injected with the unrelated Rs86 dsRNA or were left uninjected. After dsRNA injection, female ticks were held in a humidity chamber for 1 day after which they were allowed to feed on sheep inoculated intravenously with *A*. *phagocytophilum* (NY18 isolate) as described before with 20 male ticks per tick feeding cell [42]. Two sheep, Sheep 11 and Sheep 15, were used with 11 cells each to feed ticks injected with gene-specific dsRNAs and the Rs86 dsRNA and uninjected controls. Ten female ticks per group were collected after 7 days of feeding and midguts and salivary glands dissected for DNA and RNA extraction using Tri Reagent (Sigma-Aldrich, St. Louis, MO, USA) following manufacturer instructions. RNA was used to characterize gene knockdown by real-time RT-PCR with respect to Rs86 control and DNA was used to characterize *A*. *phagocytophilum* infection by PCR [16]. Remaining ticks were allowed to feed until full engorgement and tick mortality and weight were determined in individual female ticks collected after feeding. Tick weight was compared between ticks injected with test genes dsRNA and Rs86 control dsRNA by Student's t-test with unequal variance (P = 0.05). The number of ticks completing feeding was compared between ticks injected with test genes dsRNA and Rs86 control dsRNA by one-tailed Fisher's exact test (P = 0.05).

### Determination of *A*. *phagocytophilum* infection by real-time PCR

*A*. *phagocytophilum* DNA levels were characterized by *msp4* real-time PCR normalizing against tick 16S rDNA as described previously [16]. Normalized Ct values were compared between ticks injected with test genes dsRNA and Rs86 control dsRNA by Student's t-test with unequal variance (P = 0.05).

### Determination of tick mRNA levels by real-time RT-PCR

The expression of selected genes was characterized using total RNA extracted from individual nymphs and/or female midguts and salivary glands. All ticks were confirmed as infected or uninfected by real-time PCR analysis of *A*. *phagocytophilum msp4* DNA in midguts and salivary glands. Real-time RT-PCR was performed on RNA samples using gene-specific oligonucleotide primers (S5 Table) and the iScript One-Step RT-PCR Kit with SYBR Green and the CFX96 Touch Real-Time PCR Detection System (Bio-Rad, Hercules, CA, USA). A dissociation curve was run at the end of the reaction to ensure that only one amplicon was formed and that the amplicons denatured consistently in the same temperature range for every sample. The mRNA levels were normalized against tick 16S rRNA and cyclophilin as described previously using the genNorm method (ddCT method as implemented by Bio-Rad iQ5 Standard Edition, Version 2.0) [16]. Normalized Ct values were compared between test dsRNA-treated ticks and controls treated with Rs86 dsRNA or between infected and uninfected ticks by Student's t-test with unequal variance (P = 0.05).

For analysis of mRNA levels in different tick developmental stages, total RNA was extracted from eggs (three batches of approximately 500 eggs each), fed and unfed larvae (three pools of 50 larvae each), fed and unfed nymphs (three pools of 15 nymphs each), and fed and unfed males and females adults tick tissues (4 ticks each) were used for real-time RT-PCR as described before but normalizing against tick cyclophilin and ribosomal protein S4 [GenBank: DQ066214] using oligonucleotide primers rsp4-F: 5’-GGTGAAGAAGATTGTCAAGCAGAG-3’ and rsp4-R: 5‘-TGAAGCCAGCAGGGTAGTTTG-3’.

### Western blot and immunofluorescence assays

Antibodies against Porin [16] and Cytochrome c (H-104: sc-7159; Santa Cruz Biotechnology, Inc. Dallas, TX, USA) were used for Western blot and immunofluorescence studies. Total proteins used for proteomics from infected and uninfected nymphs (2 μg from each sample) were methanol/chloroform precipitated, resuspended in Laemmli sample buffer and separated on a 15% SDS-PAGE gel under reducing conditions. After electrophoresis, proteins were transferred to nitrocellulose membranes (Bio-Rad, Hercules, CA, USA), blocked with SuperBlock blocking buffer in TBS (Thermo Scientific) and incubated overnight at 4°C with rabbit polyclonal anti-Porin (dilution 1:1000) or anti-Cytochrome c (dilution 1:200) antibodies. To detect the antigen-bound antibody, membranes were incubated with goat anti-rabbit IgG conjugated with horseradish peroxidase (dilution 1:10,000; Sigma-Aldrich). Immunoreactive proteins were detected by chemoluminescence using the SuperSignal West Pico chemoluminescent substrate (Thermo Scientific), visualized with an ImageQuant 350 Digital Imaging System (GE Healthcare, Pittsburgh, PA, USA), quantified using the ImageQuant TL 7.0 software (GE Healthcare) and normalized against total proteins. Normalized protein levels (N = 2) were compared between samples by χ^2^ test (p = 0.05). Positive controls (C+) corresponded to recombinant *I*. *scapularis* Porin expressed in *Escherichia coli* (5 μg) and human HL60 cells for Porin and Cytochrome c Western blots, respectively.

For immunofluorescence, adult ticks were infected with *A*. *phagocytophilum* as described before. Female ticks were removed from the sheep 7 days after infestation, held in the humidity chamber for 4 days and fixed with 4% paraformaldehyde in 0.2M sodium cacodylate buffer, dehydrated in a graded series of ethanol and embedded in paraffin. Sections (4 μm) were prepared and mounted on glass slides. The paraffin was removed from the sections with xylene and the sections were hydrated by successive 2 min washes with a graded series of 100, 95, 80, 75 and 50% ethanol. The slides were treated with Proteinase K (Dako, Barcelona, Spain) for 7 min, washed with PBS and incubated with 3% bovine serum albumin (BSA; Sigma-Aldrich) in PBS for 1 h at room temperature. The slides were then incubated for 14 h at 4°C with primary antibodies diluted 1:100 to 1:300 in 3% BSA/PBS and after 3 washes in PBS developed for 1 h with goat-anti-rabbit IgG conjugated with FITC (Sigma-Aldrich) (diluted 1:160 in 3% BSA/PBS). The slides were washed twice with TBS and mounted in ProLong Antifade reagent (Molecular Probes, Eugene, OR, USA) or in mounting medium containing DAPI (Vector Laboratories, Peterborough, UK). The sections were examined using a Leica SP2 laser scanning confocal microscope (Leica, Wetzlar, Germany). Sections of uninfected ticks and IgGs from preimmune serum were used as controls.

### Fatty acid synthase (FAS) inhibition in cultured tick cells

The *I*. *scapularis* ISE6 tick cell line (provided by U.G. Munderloh, University of Minnesota, USA) was cultured in L15B300 medium and inoculated with the human NY18 isolate of *A*. *phagocytophilum* propagated in HL-60 cells as described previously [16]. Uninfected cells were cultured in the same way, except with the addition of 1 ml of culture medium instead of infected cells. Uninfected and infected cultures (three independent cultures with approximately 5x10^5^ cells each) were seeded in 24 well plates and treated with FAS inhibitor Cerulenin (Santa Cruz Biotechnology, Heidelberg, Germany) at 0, 5, 10 and 20 μg/ml and sampled at 0 h and 48 h after treatment. Apoptosis was measured by flow cytometry using the Annexin V-fluorescein isothiocyanate (FITC) apoptosis detection kit (Immunostep, Salamanca, Spain) following manufacturers protocol. It detects changes in phospholipid symmetry analyzed by measuring Annexin V (labelled with FITC) binding to phosphatidylserine, which is exposed in the external surface of the cell membrane in apoptotic cells. Cells were stained simultaneously with the non-vital dye propidium iodide (PI) allowing the discrimination of intact cells (Annexin V-FITC negative, PI negative), early apoptotic cells (Annexin V-FITC positive, PI negative), late apoptotic/necrotic cells (Annexin V-FITC positive, PI positive) and dead cells (Annexin V-FITC negative, PI positive). All samples were analyzed on a FAC-Scalibur flow cytometer equipped with CellQuest Pro software (BD Biosciences, Madrid, Spain). The viable cell population was gated according to forward-scatter and side-scatter parameters. The percentage of apoptotic cells (including early apoptotic, late apoptotic/necrotic and dead cells) was determined by FACS after Annexin V-FITC and PI labeling. Total DNA was extracted from 200 μl of a tick cell suspension using the RealPure Spin Kit (Durviz, Valencia, Spain) following the manufacturer's recommendations. *A*. *phagocytophilum* DNA levels were characterized by *msp4* real-time PCR normalizing against tick 16S rDNA as described before [16]. The percent of apoptotic cells and normalized *A*. *phagocytophilum* DNA levels were compared between cells analyzed at 0 and 48 h of Cerulenin treatment and/or bacterial infection by Student's t-test with unequal variance (P = 0.05; N = 3).

### JAK/STAT inhibition in cultured tick cells

The *I*. *scapularis* ISE6 tick cells were cultured and infected with the human NY18 isolate of *A*. *phagocytophilum* as described above. Infected cells were treated with 400 nM of the pan JAK inhibitor (GLPG0634; MedChem Express, New Jersey, USA), 9.2 μM of the STAT3 inhibitor (Cryptotanshinone; MedChem Express, New Jersey, USA) or a combination of both at the same concentration. Control cells were incubated culture medium alone. The inhibitors were added at the same time as the bacteria and then sampled at 48 h to extract total DNA to determine *A*. *phagocytophilum* DNA levels as described above. *A*. *phagocytophilum* DNA levels were compared between treated and control cells by Student's t-test with unequal variance (P = 0.05; N = 4).

### Phylogenetic analysis of *I*. *scapularis* FAS sequences

FAS amino acid sequences were aligned with MUSCLE (v3.7) configured for high precision [54] and the ambiguous regions were removed with Gblocks (v0.91b) [55]. The phylogenetic tree was reconstructed using the maximum likelihood method implemented in PhyML (v3.0 aLRT) [56, 57]. Internal branch confidence was assessed by the bootstrapping method (1000 bootstrap replicates). Graphical representation and editing of the phylogenetic tree were performed with TreeDyn (v 198.3) [58].

## Supporting Information

S1 Fig Correlation between differential gene expression and protein representation. (A) The analysis was done with proteins showing an infected/uninfected -4>Log2-fold>4 ratio (P<0.05) and matching *I*. *scapularis* differentially expressed transcripts (P<0.05) in any of the samples (N = 9). (B) The analysis was done with proteins showing an infected/uninfected 1>Log2-fold>-1 ratio (P<0.05) and matching *I*. *scapularis* differentially expressed transcripts (P<0.05) in any of the samples (N = 18). Normalized infected/uninfected Log2-fold values were plotted for proteome and transcriptome data and the lineal correlation curve determined. The correlation coefficient (R^2^) is shown.
(TIF)

S2 Fig Tissue-specific effect of *A*. *Phagocytophilum* infection on tick biological processes. (A) Representation of biological processes in differentially expressed genes in infected nymphs, adult female midguts and salivary glands. (B) Representation of biological processes in differentially represented proteins in infected nymphs, adult female midguts and salivary glands.
(TIF)

S3 Fig Tissue-specific effect of *A*. *phagocytophilum* infection on tick molecular function. (A) Representation of molecular function in differentially expressed genes in infected nymphs, adult midguts and salivary glands. (B) Representation of molecular function in differentially represented proteins in infected nymphs, adult midguts and salivary glands.
(TIF)

S4 Fig Tissue-specific biological processes represented in highly differentially expressed genes and represented proteins in response to *A*. *phagocytophilum* infection. (A) Representation of biological processes in highly differentially expressed genes in infected nymphs, adult midguts and salivary glands. (B) Representation of biological processes in highly differentially represented proteins in infected nymphs, adult midguts and salivary glands. Highly differentially expressed genes were selected as those with more than 50-fold (log2 normalized fold change > 5.64) difference between infected and uninfected ticks. Highly differentially represented proteins were selected as those with more than 5-fold (log2 normalized fold change > 2.32) difference between infected and uninfected ticks.
(TIF)

S5 Fig Tissue-specific molecular function represented in highly differentially expressed genes and represented proteins in response to *A*. *phagocytophilum* infection. (A) Representation of molecular function in highly differentially expressed genes in infected nymphs, adult midguts and salivary glands. (B) Representation of molecular function in highly differentially represented proteins in infected nymphs, adult midguts and salivary glands. Highly differentially expressed genes were selected as those with more than 50-fold (log2 normalized fold change > 5.64) difference between infected and uninfected ticks. Highly differentially represented proteins were selected as those with more than 5-fold (log2 normalized fold change > 2.32) difference between infected and uninfected ticks.
(TIF)

S6 Fig Validation of RNAseq and proteomics data. (A) Ten nymphs and adult female ticks were collected after feeding on infected and uninfected sheep. All ticks were confirmed as infected or uninfected by PCR. The expression of selected genes was characterized by real-time RT-PCR using total RNA extracted from individual nymphs and adult female midguts and salivary glands. The mRNA levels were normalized against tick 16S rRNA and cyclophilin, represented as infected/uninfected Log2-fold ratio (Ave+SD) and compared between infected and uninfected ticks by Student's t-test with unequal variance (*P≤0.05). (B) Differential expression of selected tick genes was compared between RNAseq and real-time RT-PCR results in nymphs (N), midguts (G) and salivary glands (SG). (C) Correlation analysis between differential expression (infected/uninfected Log2-fold ratio) values obtained by real-time RT-PCR (X values) and RNAseq (Y values). The correlation coefficients (R^2^) are shown. (D) Western blot analysis of the same protein preparations used for proteomics from uninfected (Unf) and infected (Inf) nymphs using antibodies against Porin and Cytochrome c (Cyt c). Positive controls (C+) included recombinant *I*. *scapularis* Porin (Ayllón et al., 2013) and proteins extracted from human HL60 cells for Porin and Cytochrome c, respectively.
(TIF)

S1 Table RNAseq statistics. (PDF)

S2 Table RNAseq results. (XLSX)

S3 Table Proteomics results. (XLSX)

S4 Table Differential expression/representation of putative apoptosis pathway genes/proteins. (PDF)

S5 Table Genes selected for expression analysis and RNAi. (PDF)
