# Supplementary figures and images for: Systems Biology of Tissue-Specific Response to Anaplasma phagocytophilum Reveals Differentiated Apoptosis in the Tick Vector Ixodes scapularis
Source: PLoS Genet. 2015 Mar 27;11(3):e1005120. doi: 10.1371/journal.pgen.1005120 (PMC4376793; doi:10.1371/journal.pgen.1005120)

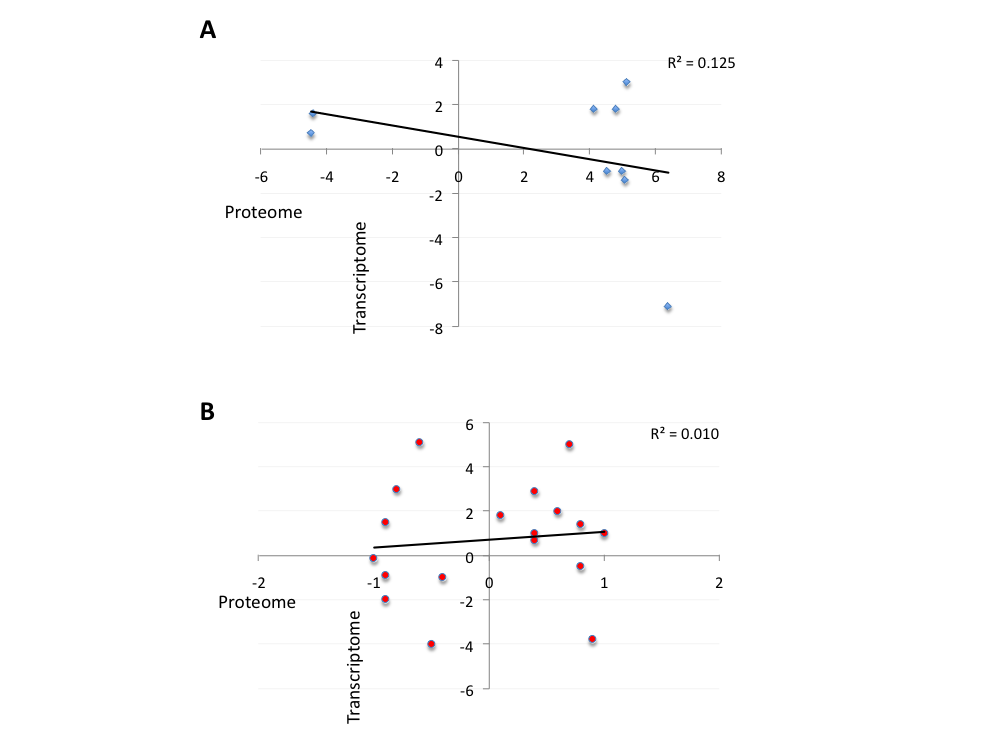

Supplement: S1 Fig — (A) The analysis was done with proteins showing an infected/uninfected -4>Log2-fold>4 ratio (P<0.05) and matching I. scapularis differentially expressed transcripts (P<0.05) in any of the samples (N = 9). (B) The analysis was done with proteins showing an infected/uninfected 1>Log2-fold>-1 ratio (P<0.05) and matching I. scapularis differentially expressed transcripts (P<0.05) in any of the samples (N = 18). Normalized infected/uninfected Log2-fold values were plotted for proteome and transcriptome data and the lineal correlation curve determined. The correlation coefficient (R2) is shown. (TIF) [file pgen.1005120.s001.tif]

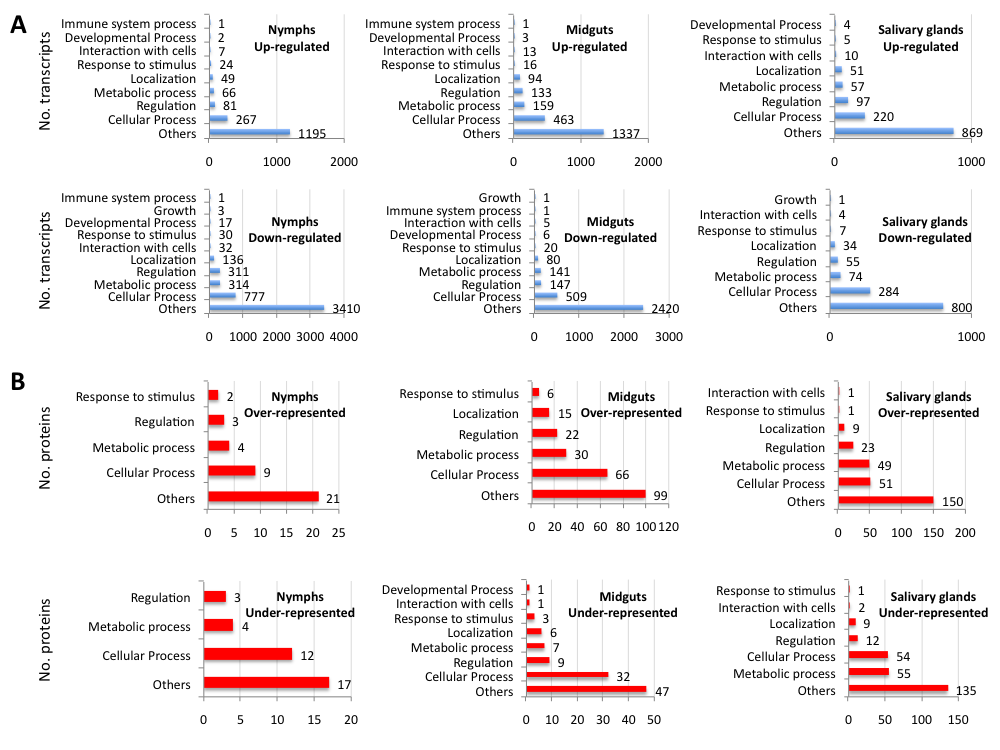

Supplement: S2 Fig — (A) Representation of biological processes in differentially expressed genes in infected nymphs, adult female midguts and salivary glands. (B) Representation of biological processes in differentially represented proteins in infected nymphs, adult female midguts and salivary glands. (TIF) [file pgen.1005120.s002.tif]

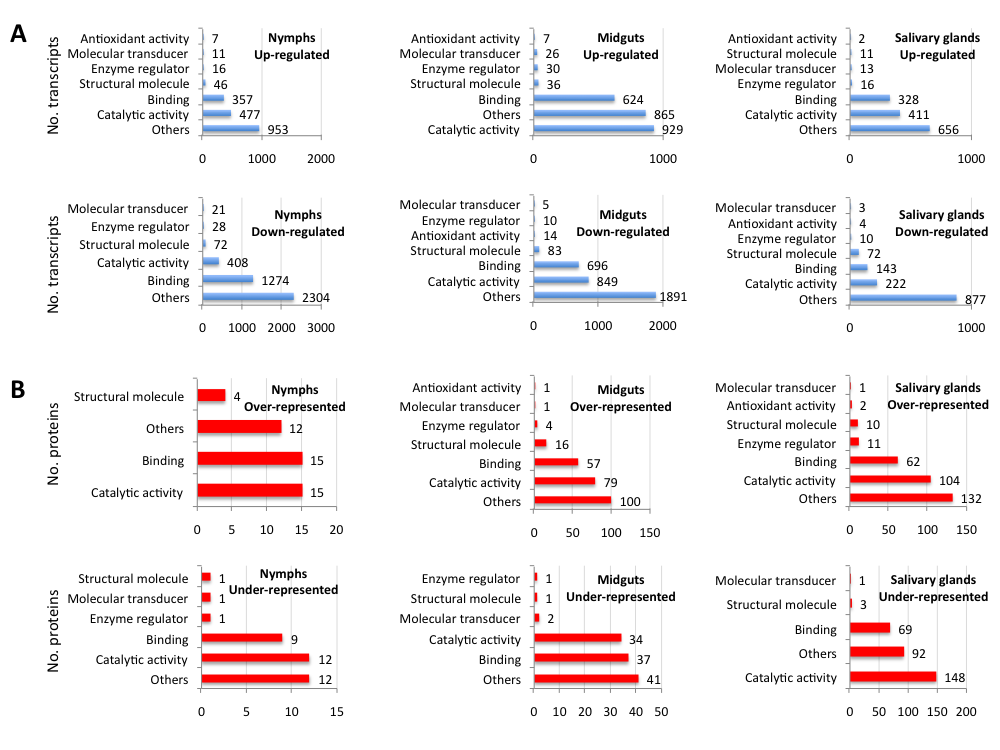

Supplement: S3 Fig — (A) Representation of molecular function in differentially expressed genes in infected nymphs, adult midguts and salivary glands. (B) Representation of molecular function in differentially represented proteins in infected nymphs, adult midguts and salivary glands. (TIF) [file pgen.1005120.s003.tif]

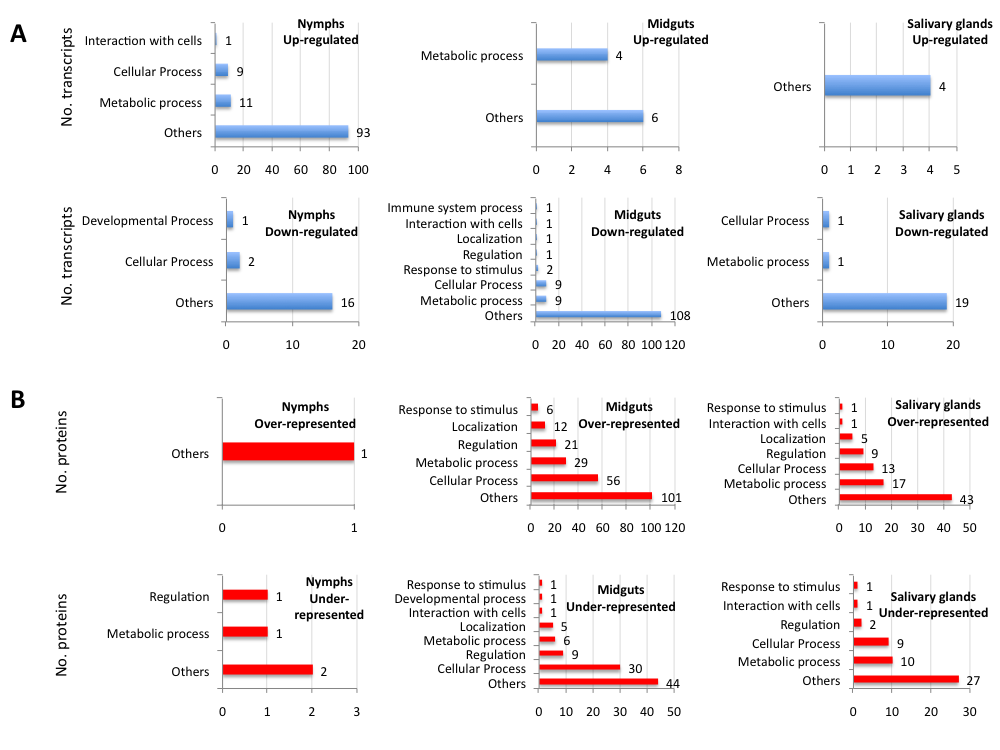

Supplement: S4 Fig — (A) Representation of biological processes in highly differentially expressed genes in infected nymphs, adult midguts and salivary glands. (B) Representation of biological processes in highly differentially represented proteins in infected nymphs, adult midguts and salivary glands. Highly differentially expressed genes were selected as those with more than 50-fold (log2 normalized fold change > 5.64) difference between infected and uninfected ticks. Highly differentially represented proteins were selected as those with more than 5-fold (log2 normalized fold change > 2.32) difference between infected and uninfected ticks. (TIF) [file pgen.1005120.s004.tif]

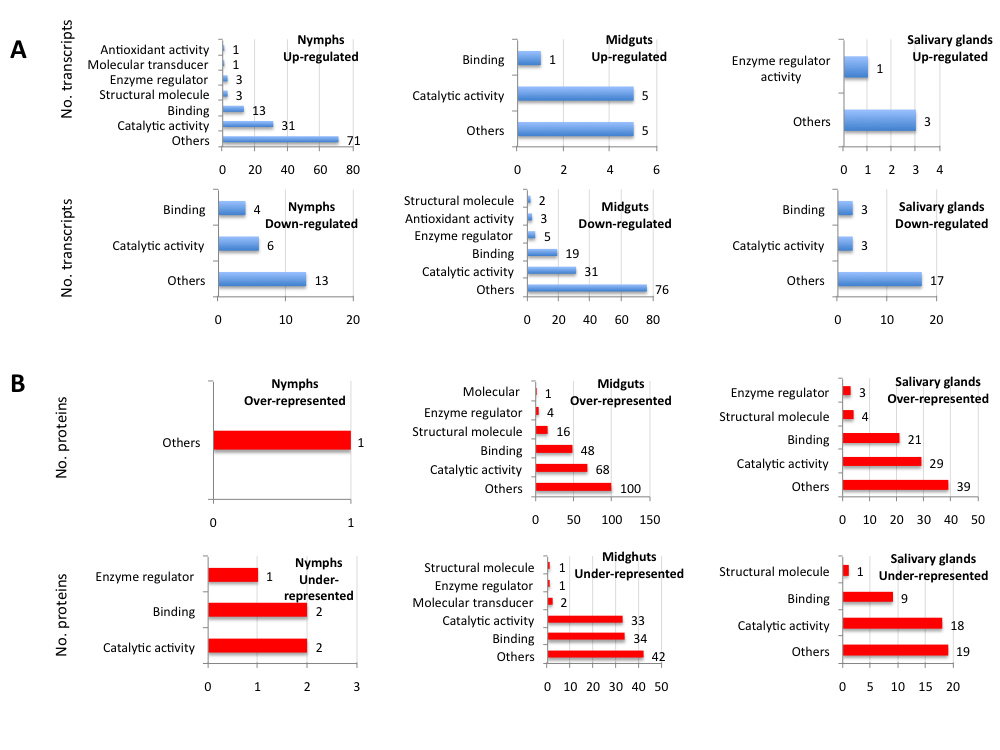

Supplement: S5 Fig — (A) Representation of molecular function in highly differentially expressed genes in infected nymphs, adult midguts and salivary glands. (B) Representation of molecular function in highly differentially represented proteins in infected nymphs, adult midguts and salivary glands. Highly differentially expressed genes were selected as those with more than 50-fold (log2 normalized fold change > 5.64) difference between infected and uninfected ticks. Highly differentially represented proteins were selected as those with more than 5-fold (log2 normalized fold change > 2.32) difference between infected and uninfected ticks. (TIF) [file pgen.1005120.s005.tif]

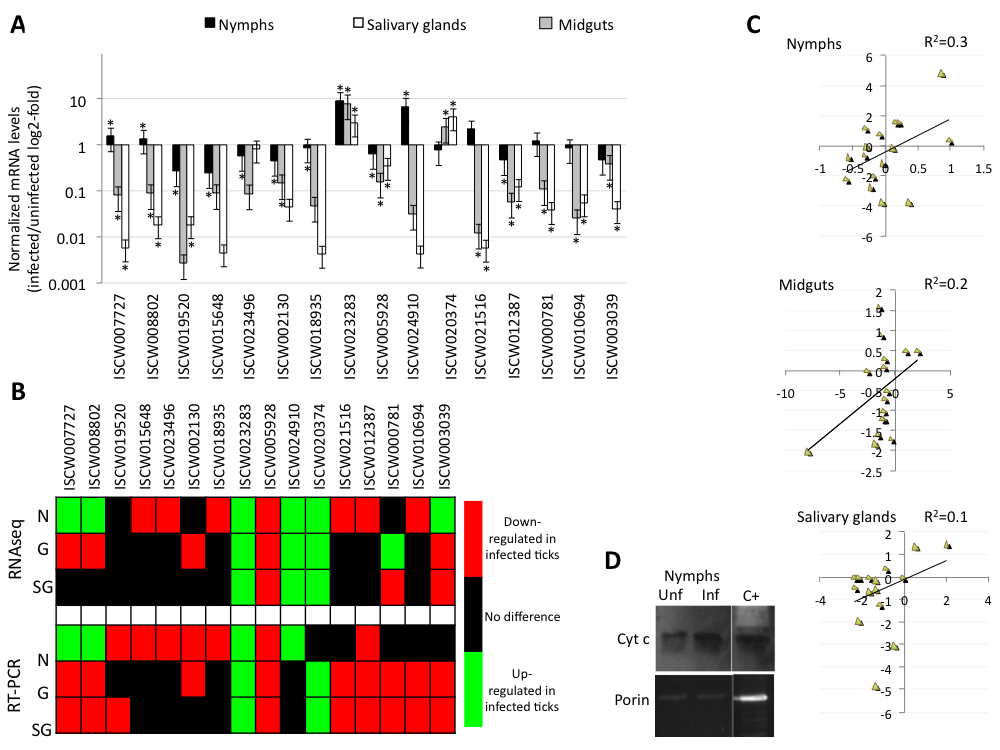

Supplement: S6 Fig — (A) Ten nymphs and adult female ticks were collected after feeding on infected and uninfected sheep. All ticks were confirmed as infected or uninfected by PCR. The expression of selected genes was characterized by real-time RT-PCR using total RNA extracted from individual nymphs and adult female midguts and salivary glands. The mRNA levels were normalized against tick 16S rRNA and cyclophilin, represented as infected/uninfected Log2-fold ratio (Ave+SD) and compared between infected and uninfected ticks by Student's t-test with unequal variance (*P≤0.05). (B) Differential expression of selected tick genes was compared between RNAseq and real-time RT-PCR results in nymphs (N), midguts (G) and salivary glands (SG). (C) Correlation analysis between differential expression (infected/uninfected Log2-fold ratio) values obtained by real-time RT-PCR (X values) and RNAseq (Y values). The correlation coefficients (R2) are shown. (D) Western blot analysis of the same protein preparations used for proteomics from uninfected (Unf) and infected (Inf) nymphs using antibodies against Porin and Cytochrome c (Cyt c). Positive controls (C+) included recombinant I. scapularis Porin (Ayllón et al., 2013) and proteins extracted from human HL60 cells for Porin and Cytochrome c, respectively. (TIF) [file pgen.1005120.s006.tif]
