# Supplementary material for: Systems Biology of Tissue-Specific Response to Anaplasma phagocytophilum Reveals Differentiated Apoptosis in the Tick Vector Ixodes scapularis
Source: PLoS Genet. 2015 Mar 27;11(3):e1005120. doi: 10.1371/journal.pgen.1005120 (PMC4376793; doi:10.1371/journal.pgen.1005120)
Supplement: S1 Table — (PDF) [file pgen.1005120.s007.pdf]

Table S1. RNAseq statistics.

Samples	No. read pairs (Sample 1 & 2)	Raw Data (Gbp) (Sample 1 & 2)	No. contigs (Sample 1 & 2)
Salivary glands from infected adult female ticks	13,957,587<br>14,133,013	2.9<br>2.9	10,308<br>10,307
Salivary glands from uninfected adult female ticks	14,088,073<br>12,987,915	2.9<br>2.7	8,702<br>8,702
Midguts from infected adult female ticks	15,103,377<br>14,633,730	3.1<br>3.0	10,964<br>10,962
Midguts from uninfected adult female ticks	10,842,475<br>11,045,326	2.2<br>2.2	11,437<br>11,437
Infected nymphs	10,457,230<br>11,122,072	2.1<br>2.2	12,950<br>12,953
Uninfected nymphs	19,943,130<br>18,856,067	4.1<br>3.8	15,560<br>15,556
