# Supplementary material for: Systems Biology of Tissue-Specific Response to Anaplasma phagocytophilum Reveals Differentiated Apoptosis in the Tick Vector Ixodes scapularis
Source: PLoS Genet. 2015 Mar 27;11(3):e1005120. doi: 10.1371/journal.pgen.1005120 (PMC4376793; doi:10.1371/journal.pgen.1005120)
Supplement: S4 Table — (PDF) [file pgen.1005120.s010.pdf]

Table S4. Differential expression/representation of putative apoptosis pathway genes/proteins.

Description	GenBank (Uniprot) accession Nos.	Differential gene expression/protein representation (Infected/Uninfected Log2-fold; P<0.05)		
		Midguts	Salivary glands	Nymphs
**Intrinsic pathway**				
Voltage-dependent anion-selective channel or mitochondrial porin	ISCW000781 (B7P5X8)	+0.3/NS	-0.5/NS	NS/NS
Hexokinase	ISCW012387 (B7QB25)	NS/NS	NS/NS	-0.6/NF
B-cell CLL/lymphoma 2 protein Bcl-2	ISCW019088 (B7PQ11)	NF/NF	NS/NF	NS/NF
	ISCW021516 (B7Q4U7)	NS/NF	NS/NS	-3.7/NF
Bcl-2-associated transcription factor BTF	ISCW003256 (B7PCD8)	-2.1/NF	NS/NF	+2.6/NF
Bcl-2 interacting protein	ISCW008101 (B7PTH9)	+1.3/NS	NF/NS	NF/NF
Apoptosis inhibitor IAP	ISCW003136 (B7PDJ3)	+1.9/NF	NF/NF	NS/NF
	ISCW015499 (B7QP11)	-1.9/NF	NS/NF	+0.4/NF
	ISCW010694 (B7Q7F6)	NS/NF	NS/NF	-3.7/NF
	ISCW014060 (B7QLC6)	NS/NF	NS/NF	+2.0/NF
	ISCW002567 (B7P7P7)	NF/NS	NF/NS	NF/NF
Cytochrome c	ISCW008740 (B7Q2Y4, Q4PM75)	+0.8/NS	-0.4/NS	+0.3/NF
Smac/DIABLO	ISCW000014 (B7P1U9)	-0.9/NF	NS/NF	+0.7/NF
	ISCW000886 (B7P5Y4)	-1.9/NF	NS/NS	+3.3/NF
HtrA2/Omi serine protease	ISCW020167 (B7PZV8)	NS/NF	NS/NS	-0.9/NF
Caspases	ISCW015329 (B7QNZ9)	NS/NS	NF/NS	+0.6/NF
	ISCW022545 (B7QAN7)	NS/NF	NS/NF	-1.6/NF
	ISCW013172 (B7QF31)	NS/NS	NS/NS	-2.5/NF
	ISCW003039 (B7PDI2)	-1.7/NS	-4.8/-2.4	+1.2/NF
Caspase-2	ISCW014750 (B7QHN6)	NS/NS	NF/NS	-2.9/NF

**Extrinsic pathway**				
Fas apoptotic inhibitory molecule	ISCW011757 (B7Q584)	-1.5/NF	NS/NF	-2.7/NF
Fatty acid synthase (FAS)	ISCW000257 (B7P631)	+4.5/-1.7	NF/-1.8	-4.3/NF
	ISCW009053 (B7PYN9)	+5.6/NS	NS/-1.7	-3.9/NS
	ISCW010397 (B7Q724)	NS/NF	NF/-1.8	-5.3/NF
	ISCW010398 (B7Q725)	NS/NF	NF/NF	-3.8/NF
	ISCW010399 (B7Q726)	NS/NF	NF/NF	-4.6/NF
	ISCW014349 (B7QKF5)	NS/NF	NF/NF	NS/NF
	ISCW014350 (B7QKF6)	NS/NF	NF/NF	NF/NF
	ISCW001913 (B7PCH5)	NF/NF	NF/NF	-3.5/NF
	ISCW014347 (B7QKF3)	NF/NF	NF/NF	-3.3/NF
	ISCW014535 (B7QGS8)	NF/NF	NF/NF	-3.1/NF
	ISCW018177 (B7PEX3)	NF/NF	NF/NF	-2.6/NF
	ISCW004392 (B7PK76)	NF/NS	NF/NF	NS/NF
	ISCW001073 (B7P2U7)	NF/NF	NF/NF	NS/NF
	ISCW014149 (B7QL04)	NF/NF	NF/-1.8	NS/NF
	ISCW018176 (B7PEX2)	NF/NF	NF/NF	NS/NF
	ISCW024925 (B7QJQ3)	NF/NF	NF/NF	-4.6/NF
	ISCW009844 (B7Q3T1)	NF/NF	NF/NF	-5.3/NF
	ISCW008122 (B7PVK8)	NF/NF	NF/NF	-4.8/NF
	ISCW014534 (B7QGS7)	NF/NF	NF/NF	-3.4/NF
	ISCW001566 (B7P4V0)	NF/NF	NF/NF	NS/NF
	ISCW005148 (B7PFR3)	NF/NF	NF/NF	NS/NF
	ISCW010394 (B7Q6N6)	NF/NF	NF/NF	NS/NF
	ISCW011309 (B7Q6F3)	NF/NF	NF/NF	NS/NF
	ISCW011310 (B7Q6F4)	NF/NS	NF/NF	NS/NF

Death receptor	ISCW015148 (B7QNQ9)	NS/NF	NS/NF	NS/NF
	ISCW006321 (B7PMY2)	NS/NF	NS/NF	-1.8/NF
**Perforin/Granzyme pathway**				
Granzyme	ISCW001322 (B7P540)	NS/NF	NF/NF	-1.3/NF
	ISCW013112 (B7QDI9)	NF/NF	NF/NF	-2.1/NF
	ISCW003779 (B7PHH4)	NF/NF	NF/NF	NF/NF
	ISCW000320 (B7P0L5)	NF/NF	NF/NF	-2.8/NF
	ISCW010999 (B7Q921)	NF/NS	NF/NF	NF/NF
	ISCW011206 (B7Q613)	NF/NF	NF/NF	NF/NF
	ISCW011961 (B7QB06)	NF/NF	NF/NF	NF/NF
**Execution pathway**				
Caspase-3	ISCW001741 (B7P6Y4)	NS/NF	NS/NS	+1.2/NF
poly ADP-ribose polymerase, PARP	ISCW019519 (B7PWC4)	NS/NS	NS/NS	-1.2/NF
	ISCW017128 (B7PA88)	-1.3/NF	+0.7/NF	+1.6/NF
	ISCW019970 (B7PV22)	NS/NS	NS/NS	-2.9/NF
Alpha fodrin, spectrin alpha chain	ISCW000012 (B7P1U8)	+1.0/NS	+0.9/NS	-1.2/NS
Caspase-activated DNase, CAD	ISCW018943 (B7PNY7)	NS/NF	NS/NF	NS/NF
Inhibitor of CAD, ICAD	ISCW017650 (B7PJN6)	NS/NF	+3.1/NF	NS/NF
**Other apoptosis-related genes**				
Apoptosis-promoting RNA-binding protein TIA-1/TIAR	ISCW014211 (B7QI53)	+1.2/NS	+1.1/NS	+1.4/NS
Programmed cell death protein	ISCW006407 (B7PMJ5)	-0.7/NS	NS/NS	+1.3/NF
	ISCW016513 (B7P6D3)	-0.9/NF	NS/NF	+0.8/NF
Bax-mediated apoptosis inhibitor TEGT/BI-1	ISCW018655 (B7PL49)	-0.7/NF	NS/NF	-0.7/NF
Hypothetical protein (mitochondrial ribosomal S23/S29 protein)	ISCW005445 (B7PNA5)	+1.4/NF	NS/NS	NS/NF

Ccar1	ISCW023286 (B7QIS5)	+2.6/NS	+1.4/NF	-0.8/NF
Apoptosis inducing factor	ISCW005256 (B7PLJ8)	+2.6/NF	NS/NS	NS/NF
Apoptosis associated protein with THAP domain	ISCW021993 (B7QFZ9)	-1.3/NF	+1.2/NF	NS/NF
Cellular tumor antigen P53	ISCW012889 (B7QF52)	-1.3/NF	NS/NF	+0.8/NF
Anamorsin homolog	ISCW019144 (B7PP17)	-2.0/NS	-1.0/NS	NS/NF
TP53-regulated inhibitor of apoptosis	ISCW006194 (B7PKG1)	-2.3/NF	-0.9/NF	+1.1/NF
Cell death regulator Aven	ISCW018605 (B7PKZ4)	-2.5/NF	NS/NF	+1.7/NF
	ISCW018605 (B7PKZ4)	-2.5/NF	NS/NF	+1.7/NF
Hypothetical protein	ISCW011866 (B7QE72)	-2.9/NF	+1.1/NF	NS/NF
Defender against cell death	ISCW024010 (B7P488, Q4PM70)	+0.8/NF	-1.2/NS	-1.5/NF
NADH:ubiquinone oxidoreductase, B16.6	ISCW005224 (B7PFN5)	+1.0/NS	NS/NS	-1.2/NF
Death-associated protein kinase DAPK	ISCW005225 (B7PFN6)	+3.4/NF	NS/NS	NS/NF
	ISCW020324 (B7PZW2)	NS/NF	+2.5/NF	+0.6/NF
Ataxin-10	ISCW022385 (B7QGH7)	-1.0/NF	NS/NS	-1.7/NF
Apoptosis antagonizing transcription factor	ISCW007430 (B7PW14)	NF/NF	NF/NF	NF/NF
THAP domain containing, apoptosis associated protein 2	ISCW024143 (B7PCQ7)	NF/NF	NF/NF	NF/NF
	ISCW024918 (B7QIT8)	NF/NF	NF/NF	NF/NF
Apoptosis-promoting RNA-binding protein TIA-1/TIAR	ISCW016601 (B7PBU8)	NF/NS	NF/NS	NF/NF
Apoptosis stimulating of P53	ISCW007014 (B7PSS5)	NF/NF	NF/NF	NF/NF
**Janus kinase/signal transducers and activators of transcription (JAK/STAT) pathway**				
JAK (Hopscotch) tyrosine protein kinase	ISCW016158 (B7P4T8)	+2.0/NF	NS/NF	-2.5/NF
STAT3	ISCW005692 (B7PKW7)	+1.2/NF	NS/NF	-1.1/NF

JAK receptor protein tyrosine phosphatase (Domeless)	ISCW016699 (B7PAS3)	+2.1/NF	NS/NF	-0.9/NF
PIAS Sumo ligase	ISCW005295 (B7PLL7)	NS/NF	NS/NF	NS/NF
SOCS box SH2 domain-containing protein	ISCW019435 (B7PTW4)	-2.2/NF	NS/NF	-1.0/NF

Abbreviations: +, up-regulated/over-represented in infected ticks; -, down-regulated/under-represented in infected ticks; NS, no significant differences between infected and uninfected ticks; NF, transcript/protein not found.
