# Supplementary material for: Systems Biology of Tissue-Specific Response to Anaplasma phagocytophilum Reveals Differentiated Apoptosis in the Tick Vector Ixodes scapularis
Source: PLoS Genet. 2015 Mar 27;11(3):e1005120. doi: 10.1371/journal.pgen.1005120 (PMC4376793; doi:10.1371/journal.pgen.1005120)
Supplement: S5 Table — (PDF) [file pgen.1005120.s011.pdf]

Table S5. Genes selected for expression analysis and RNAi.

Gene ID	Forward and Reverse Primers for dsRNA synthesis (5´- 3´)*	Forward and Reverse Primers for real-time RT-PCR (5´- 3´)
ISCW007727	Not used for RNAi	GGCGTCAGGGCTACTGTATT<br>ACAACAAGCTGCGTGAATGC
ISCW008802	Not used for RNAi	ATGTGGACATTCCTCTGGCC<br>TCTCTCCAGCAGCTCCAGAT
ISCW019520	Not used for RNAi	TGATGCACCACCAGGGAAAG<br>ACACTGGATGCCCAGACTTG
ISCW015648	Not used for RNAi	CGACTCAAACAGGCTCAGGT<br>TCTCGCTGTTGAACACCTCC
ISCW023496	Not used for RNAi	CGGAGATCTGCCTGCTCAAA<br>GCTCTGCTGTCGACTCTTCA
ISCW002130	Not used for RNAi	GGAAGATCTGGAGCGTTGGT<br>CTCGGCATTTACCGAGTCGT
ISCW018935	Not used for RNAi	GAGGACTGCTGCTACGTCAC<br>ACCAGGTTCAGGTTCAGCTC
ISCW023283	Not used for RNAi	AGCGGCCAACATTCGGGAGG<br>ATGAGCCCCACCTGGCGGAA
ISCW005928	Not used for RNAi	GCTACCACAACTAGAAGAAACC<br>TGAAGCAACGTAAGGAGTTT
ISCW024910	Not used for RNAi	GACACTAGCGAGAACATCCGA<br>ACACTTGGATCGTGACGGC
ISCW020374	Not used for RNAi	AGGCCAAGGCTGCTTCCAC<br>CGACACGGTGCCCTCGAC
ISCW021516 (Bcl-2)	CGTCAACGAGACCGACAAGA<br>TGGCTCAATGAAAAACGCCG	TTTGCTCGCATACCGAGGTC<br>TACGACGAGATGCACGCCACA
ISCW012387 (Hexokinase)	CTCGCCCCTAAGAAACCGTT<br>TGAAACGTATGGCTGCAGGT	CATCTGCCTCGCTGAATTGC<br>AACGGTTTCTTAGGGGCGAG
ISCW000781 (Porin)	ATGGCTCCTCCGTGCTACG<br>AATTGTTGACCTTCGCCCT	GTCGTGAAACTCGACTGCAA<br>CCGTGTTCCACTTCTCCTTC
ISCW010694 (IAP)	CAAGGCGGGCTTCTTCTACA<br>GAGGAGGGCTGGTGAAAGTC	AACTCCCACTTGAAGATGGC<br>CATGGTCGGAGACACCTGG
ISCW003039 (Caspase)	AAGACATCGCTCGAGTCACC<br>CCCACGAAAACGTCCGGATA	CATCAACGCCTTGTGCTCAG<br>AAGCTGCACTGTCTCGTGAT
ISCW008740 (Cytochrome c)	Not used for RNAi	AGAGCAGCCATGGTCGAAAT<br>TCTGTGTAGCTGAAGCCTGC

*All primers contained T7 promoter sequences at the 5´-end (5’-TAATACGACTCACTATAGGGTACT-3’ or 5’-TAATACGACTCACTATAGGGAG-3’).
